# Functionally important segments in proteins dissected using Gene Ontology and geometric clustering of peptide fragments

**DOI:** 10.1186/gb-2008-9-3-r52

**Published:** 2008-03-10

**Authors:** Karuppasamy Manikandan, Debnath Pal, Suryanarayanarao Ramakumar, Nathan E Brener, Sitharama S Iyengar, Guna Seetharaman

**Affiliations:** 1 Bioinformatics Centre, Indian Institute of Science, Bangalore 560012, India; 2 Department of Physics, Indian Institute of Science, Bangalore 560012, India; 3 Supercomputer Education and Research Centre, Indian Institute of Science, Bangalore 560012, India; 4 Department of Computer Science, Louisiana State University, Baton Rouge, LA 70803, USA; 5 Main correspondence

## Abstract

We have developed a geometric clustering algorithm using backbone φ,ψ angles to group conformationally similar peptide fragments of any length. By labeling each fragment in the cluster with the level-specific Gene Ontology 'molecular function' term of its protein, we are able to compute statistics for molecular function-propensity and *p*-value of individual fragments in the cluster. Clustering-cum-statistical analysis for peptide fragments 8 residues in length and with only *trans *peptide bonds shows that molecular function propensities ≥20 and *p*-values ≤0.05 can dissect fragments within a protein linked to the molecular function.

## Abstract 2

A geometric clustering algorithm has been developed to dissect protein fragments based on their relevance to function.

## Background

Analysis of the protein fold reveals only a part of the information contained in the protein structure, whereas analysis of protein structure as an assembly of peptide fragments in a defined order provides additional information with respect to certain desired features [1-4]. Simple analysis of the distribution of fragments and their recurrence in protein structures helps to better understand the underlying rules of their formation [5,6]. Since structure is better conserved during evolution than sequence, structural similarities help to more effectively identify remote evolutionary relationships. They can be reliably used in identifying functional sites as well as functions of proteins on a larger scale [7].

Protein annotation efforts benefit immensely from knowledge of functional signatures in primary, secondary and tertiary structures. Calcium-binding motifs, such as the EF hand [8] and zinc-binding [9], chitin-binding [10] and ATP/GTP-binding motifs [11], are well known examples of fragment-based functional three-dimensional structural signatures in proteins. Interestingly, however, only a few fragment-based geometric clustering methods exist that can automatically identify motifs and relate them to function [12]. The lack of such methods is mainly due to the large computation time required to perform the studies. To bypass such difficulties, some authors have used clustering of the secondary structure patterns [13] or symbolic representation of structural fragments [14-16] to relate protein fragments to function. In most cases the studies are limited to describing the known relevance of fragments in inferring biochemical function. This is in contrast to a large number of methods developed for finding functionally significant three-dimensional motifs formed from non-contiguous amino acids in the polypeptide chain. Structure-based residue/chemical group clustering in combination with multiple sequence alignment has been frequently used for this purpose [17-19]. Numerous studies also exist where sequence information alone has been used to assess function [20]. One such recent study [21] identifies function-associated loops in proteins using Gene Ontology (GO) [22] molecular function (MF) terms. In this case, the starting information was structure, and from that the sequence pattern was derived.

Fragments derived from structure-based sequence signatures offer an attractive way to annotate protein function because of their applicability to both sequences and structures with unknown function. In this paper we have used a clustering algorithm based on backbone φ,ψ torsion angles to find conformationally similar peptide fragments of different lengths from the FSSP library [23], which contains a large number of proteins with distinct folds. This algorithm is derived from the demographic clustering technique used in data mining applications [24]. A distinct feature of the clustering procedure ensures that the clusters are formed with their centers at the locations with the densest distributions of points in the torsion angle space. The clusters show that protein fragments extremely divergent in sequence can adopt similar conformations. Yet within the clusters, GO MF terms associated with the fragments (as derived from the Protein Data Bank (PDB) annotation) can be over-represented, and identified by a statistically significant distribution of propensity values, highlighting the primary importance of the fragment to biochemical function. Geometric and sequence signatures derived from this work will be useful in assessing proteins with unknown function. Protein modeling, design and engineering experiments would also benefit from this work.

## Results

### Fragments used in clustering

**Figure 1:**
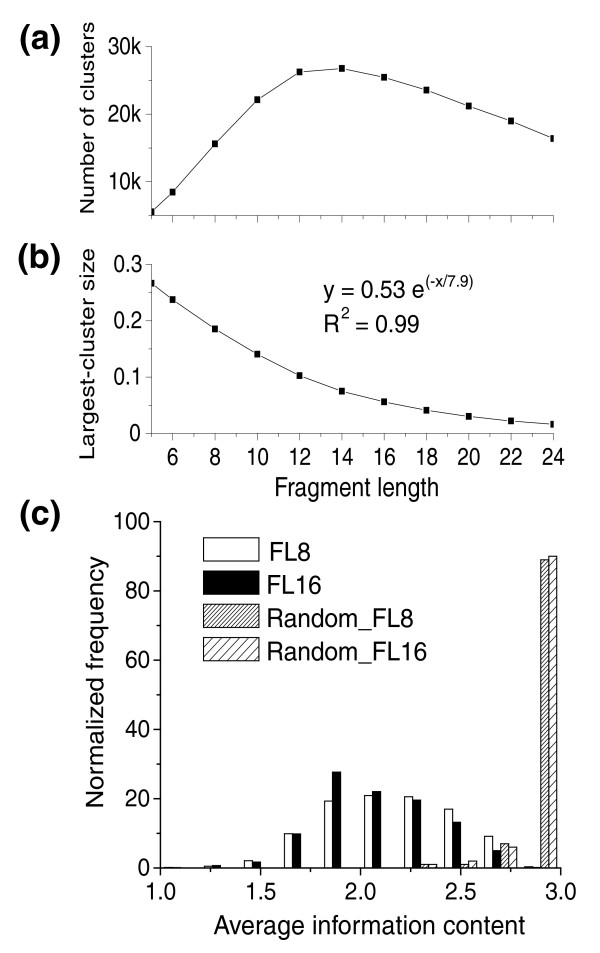
Plot showing **(a) **the variation of the number of clusters (≥2 fragments) with fragment length, **(b) **the variation of the largest cluster size (expressed as a fraction of the total number of clustered fragments in the database) with fragment length, and **(c) **the distribution of average information content of all clusters. Data are plotted for clusters with ≥10 fragments.

The clustering algorithm was applied to 2,619 PDB [25] chains culled from the FSSP database, each representing a unique fold as given in the DALI domain dictionary (see Additional data file 1 for PDB details). We clustered peptide fragments of various lengths that contained only *trans *peptide bonds; Table 1 lists the statistics for lengths 5-24, which we used for this study. A maximum of 455,305 fragments with a length of 5 residues were generated from all the PDB chains; this number decreased linearly with increasing fragment length (FL; number of fragments = (-13,243 × FL) + 468,104; R^2 ^= 0.99). The largest number of clusters with 2 or more fragments were generated for the data set including fragments with a FL of 14 (data set FL14; 26,778 clusters). The number of clusters varies non-linearly with increasing FL (Figure 1a). For the FL5 data set, the number of clusters, as well as the number of singletons left unclustered, is low. With increasing FL up to 14, the number of clusters increases, as does the number of singletons left unclustered. As a result, the sequence diversity of fragments is high in low FL clusters compared to high FL clusters. Indeed, the largest cluster size for at a FL of 5 constitutes 27% of the total FL5 data set (Table 1). The fraction of total data points included in the largest cluster decreases exponentially with increasing FL (Figure 1b). When we use all clusters with 2 or more members, 98.8% of the total fragments in the database are clustered for *trans *FL5. The coverage progressively decreases to below 40% for *trans *FL20 or more. If we consider only clusters with 10 or more fragments, at least 40% coverage can be achieved with FLs of only 14 or less. The compactness of clusters also increases with increasing FL (Table 1, last column). Representative distributions for FL8 and FL16 across all clusters also show similar trends (Additional data file 2). These suggest that the optimal range for scanning biologically relevant motifs is between FLs of 8 and 14, where we can choose large clusters ignoring short fragments and also eliminate a large number of clusters with just a few members. To identify what cluster size is significant for statistical analysis, we plotted the normalized frequency of occurrence of the clusters from individual FL data sets (data not shown) against the rank of clusters in terms of size. The distribution follows a power-law and the distribution of clusters of both FL8 and FL16 with ten or more fragments follow Zipf's law, suggesting their suitability for data mining analysis [26].

**Table 1:** Overall statistics of generated clusters from all *trans *fragments

FL	Total fragments	Total number of clusters with >2 fragments (% fragments clustered)	Largest cluster	
			
			Size (% of total fragments)	Compactness* (SD)
5	455,305	5,544 (98.8)	121,220 (27)	2.92 (1.8)
6	446,479	8,466 (97.3)	106,020 (24)	2.62 (1.5)
8	429,793	15,617 (92.1)	79,646 (19)	2.23 (1.2)
10	414,207	22,120 (83.7)	58,150 (14)	2.0 (1.0)
12	399,615	26,228 (72.9)	40,935 (10)	1.81 (0.87)
14	385,866	26,778 (61.2)	28,313 (7)	1.68 (0.77)
16	369,760	25,455 (50.8)	19,469 (5)	1.56 (0.70)
18	360,537	23,302 (41.2)	13,519 (4)	1.45 (0.63)
20	348,824	21,079 (33.4)	9,551 (3)	1.37 (0.59)
22	337,679	18,646 (28.8)	6,804 (2)	1.29 (0.55)
24	327,010	16,132 (21.4)	4,966 (2)	1.22 (0.52)

*(Average of the distances of all fragments in a cluster from its center)/(2 × FL). SD, standard deviation.

### Information content of clustered fragments

Before performing any analysis with the clusters, we also checked their distribution of average information content (sequence entropy). As can be seen in Figure 1c, for a given cluster, the more the fragment pairs have the same residues at identical positions, the lower the information content. The major peaks of the distribution of information content derived from geometric clusters are at values higher than 1.0 for both FL8 and FL16. Some of the clusters with large information content (>2.0) have an especially large number of fragments with extensive sequence diversity. Further analysis showed that only clusters with less than ten fragments, which also did not conform to Zipf's law, had information contents <1.0. A general survey of FL8 clusters with 10 or more fragments showed only 592 of them having at least one position with greater than 80% amino acid conservation. Notably, 97% of the conserved residues were found to be Gly and the remaining conserved residues are Cys, Asp, Lys and Ser in decreasing order. However, the overall distribution of amino acids between the clustered fragments and the total data set of proteins was found to be similar, indicating the data set used for this study is unbiased. Analysis with FL16 clusters essentially gave similar results (Figure 1c), with Gly again being the most conserved residue followed by Asp and Lys.

### Identification of functionally important fragments

**Figure 2:**
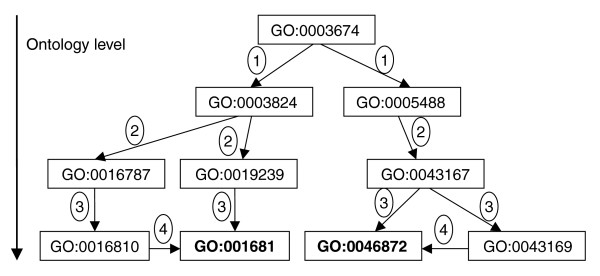
Figure depicting the concept of the GO directed acyclic graph for PDB entry 1woh. Each node is represented by a unique GO MF term (GO:0003674, molecular function; GO:0003824, catalytic activity; GO:0005488, binding; GO:0016787, hydrolase activity; GO:0016810, hydrolase activity, acting on carbon-nitrogen (but not peptide) bonds; GO:0016813, hydrolase activity, acting on carbon-nitrogen (but not peptide) bonds, in linear amidines; GO:0019239, deaminase activity; GO:0043167, ion binding; GO:0043169, cation binding; GO:0046872, metal ion binding). The level of each GO term is indicated in the round text box. Note that the same GO term can have multiple levels depending on how you trace the path to the root GO term. The terms depicted in bold are annotated for the PDB in the GOA database [68]. A protein can be represented at various GO levels by taking the parent GO terms of the original PDB annotation.

In order to identify the functional relevance of the fragments in clusters, we investigated the GO MF terms of the fragments in clusters mapped from their original PDB annotations. It was found that many of the functionally significant structural motifs grouped into distinct clusters, for example, helix-turn-helix DNA binding, ATP/GTP binding P-loop, iron binding motifs and so on. However, we did not find any cluster that had only a single GO term across all clustered fragments. This was because in many cases similar GO terms from different levels in the GO graph were present as the annotated term (Figure 2). Therefore, to cluster GO terms in order to identify functionally significant fragments within the cluster that relate directly to the function of the protein, it was important to map the original GO MF (as available from the PDB) terms of the fragments to a specific level in the Ontology graph. It should be noted that a GO term can have multiple levels depending on how its path to the root GO term in the Ontology graph is traced. The 678 and 657 unique GO MF terms obtained from the PDB for clustered fragments of FL8 and FL16, respectively, were used for mapping the GO terms to minimum ontology levels of 3, 4, and 5. In some cases, however, a fragment originally PDB annotated at level 3 could not be represented at a deeper level 5 based on the Ontology graph. Therefore, although we have done our calculations for all the levels, because of poorer coverage at deeper levels we discuss the details of results available from only level 3.

**Figure 3:**
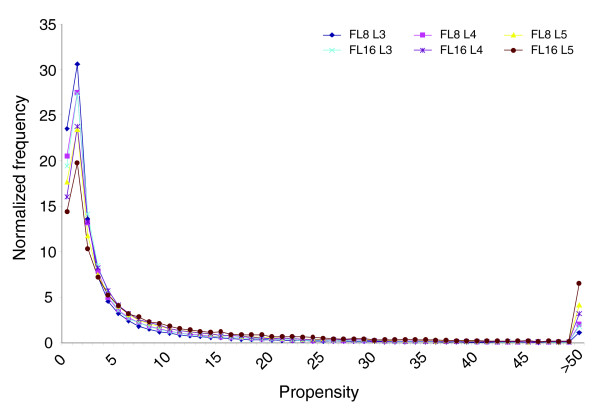
Distributions of propensity values of GO MF terms computed in each cluster. L3, L4, and L5 refer to ontology levels 3, 4 and 5, respectively.

The counts of GO MF terms mapped at levels 3, 4, and 5 for fragments in each cluster were used to calculate the propensity of occurrence of the unique GO terms in each cluster. The distributions of propensity values are shown in Figure 3. It can be seen that the fraction of fragments with propensity values 0-4 is higher at level 3 for both FL8 and FL16, decreasing gradually for levels 4 and 5. The occurrence of propensity-values shows a peak between 1 and 2 and follows a normal distribution with an extended tail beyond propensity value 5 or more. Till this point a Gaussian function can be fit to all the curves with least-square (R^2^) values >0.9. Interestingly, a propensity value different from 1 itself points to its statistical significance; but by plotting the distribution we further find that fragments with GO terms with propensity values beyond 5 are enriched to have a significant functional relevance. Using the hypergeometric distribution, we further confirmed the statistical significance by calculating *p*-values for FL8 and FL16 fragments for all GO terms mapped to levels 3, 4 and 5. For all GO terms, when we examine the distribution of *p*-values against propensity, we clearly see that for *p*-values ≤0.05 the propensity values are always ≥20 (data not shown). Therefore, we retained these statistically significant high propensity fragments for further analysis.

Since fold is intimately related to function, we also asked if we get similar results when we repeat our calculations, replacing the GO terms with CATH database [27] identifiers for the proteins. We mapped GO-based and CATH-based (four level hierarchy) propensities for individual fragments in our data set, wherever both GO term and CATH identifiers were present for the protein. The results showed poor correlation between CATH-based and GO-based propensities (correlation coefficient = 0.13). When we considered only fragments with GO-based propensity ≥20, the correlation improved marginally to 0.18. This indicated that the information available from fold-based propensity and GO term-based propensity is distinct.

### Relation to PROSITE patterns

To verify if indeed GO-based propensity indicated meaningful inference of functional relevance, we selected 1,797 fragments with propensity values ≥20 from the FL8 clusters (Table 2; see Materials and methods for selection protocol). The relevance of a fragment to function was probed by examining if the fragment overlaps with a PROSITE [28] pattern. The criteria of presence/absence, overlap/non-overlap of PROSITE patterns allowed grouping into four categories for each protein fragment. The first group (Group 1) is where the protein does not have any PROSITE signature and possibly the fragment derived sequence pattern can be used as a new regular expression signature pattern. In the second group (Group 2), the protein has one or more PROSITE pattern(s), but the sequence of the fragment does not overlap with them. In the remaining two cases (Groups 3 and 4), the PROSITE pattern either overlaps partly or contains the sequence of the fragment. As can be seen, a large number of patterns were predicted from Groups 1 and 2, which constitutes new information. To establish the functional importance of these fragments, we randomly picked them for literature review. All the randomly chosen fragments we reviewed were identified to be functionally important, representative examples [29-42] of which are listed in Table 3. The *p*-values were ≤0.05 in all cases, indicating statistical significance. These suggested that a GO MF based analysis of propensities and associated *p*-values allows a strong relation of fragments to relevant biochemical functions. While reviewing the literature we checked if the relevance of a fragment to the function of the protein was evident from the text, explaining a direct relationship to experimentally determined known functional sites in proteins. A recheck of the results with FL16 fragments using level 3 GO MF terms showed occasional overlap with FL8 results, indicating that results common to both the fragment lengths may be suitably used to enhance the confidence of interpretation, wherever possible. In general, the number of high propensity fragments for a protein may vary widely, but larger proteins tend to have more of them.

**Table 2:** The distribution of selected FL8-derived sequence patterns with propensity ≥20

Group number	Occurrence of the sequence pattern	Number of patterns/PDB entries
1	No PROSITE pattern for the protein	521/50
2	The sequence occurs outside the PROSITE pattern	838/106
3	The sequence is within the PROSITE pattern	364/76
4	The sequence overlaps with the PROSITE pattern	107/35

See Materials and methods for the method of selection.

**Table 3:** Details of arbitrarily chosen FL8 fragments with propensity ≥20 mapped from GO propensity calculations at level 3

GO MF	Propensity	PDB entry [reference]*	Start^†^	Functional description	*P*-value
0004016	1,816	1azsA [34]	489	VC1 and IIC2 domain interface	0.0006
0019210	1,450	1jsuC [35]	61	Highly conserved β hairpin from p27 interacting with Cdk2 and inhibiting the cyclin-Cdk2 complex	0.0007
0000036	685	1t8kA [33]	19	Part of ligand binding region	0.0014
0016638	450	2bbkL [36]	48	Involved in protein-protein interactions	0.002
0042030	395	1n7lA [32]	13	Important loop connects two helices	0.002
0016566	382	1dvoA [31]	148	Part of large negatively charged region for RNA binding	0.003
0004016	168	1azsA [34]	501	Part of binding pocket of FKP^‡^	0.006
0004879	149	1ie9A [37]	288	Forms part of active site pocket	0.007
0016813	137	1wohA [30]	272	One of the active site residues is present	0.007
0016247	107	1oaw [38]	30	Conserved cysteines are present	0.009
0004930	98	1ijyA [29]	113	Surface exposed loop with conserved 'WP' sequence	0.01
0004383	92	1xbnA [39]	74	Forms part of HEM binding pocket	0.01
0005158	61	1qqgB [40]	56	Part of a cationic cluster^§^	0.02
0008428	61	1b2uD [41]	39	Interact with the active site residues	0.02
0003724	26	1fukA [42]	341	Conserved interaction with DEAD box motif	0.04

*These proteins do not have a PROSITE sequence signature. The chain identifier is given after the four letter PDB code, wherever present. ^†^Residue number as given in PDB. ^‡^Only PROSITE domain signature exists: 391-518. ^§^Only PROSITE domain signature exists: 12-114.

### Examples of sequence-structure patterns

#### Group 1: NS3 protease

**Figure 4:**
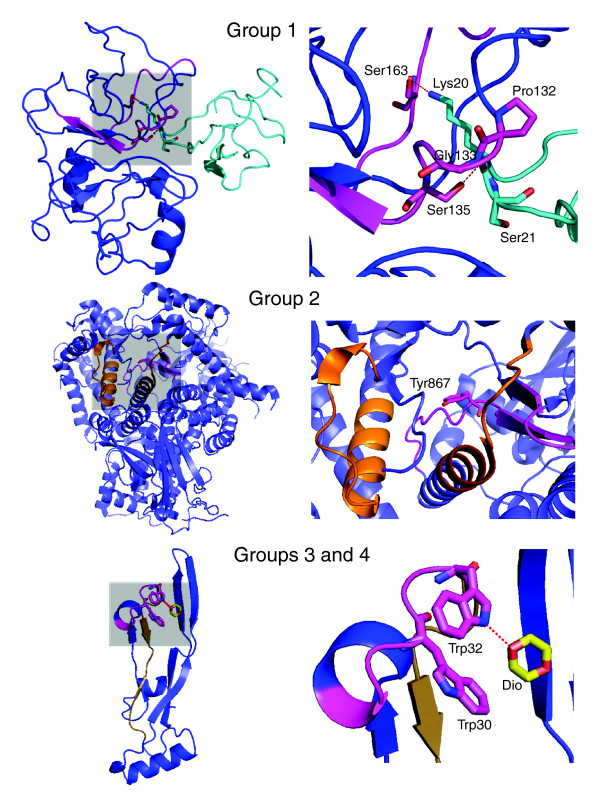
Representative examples from different groups of predictions obtained from our clustering method (see Table 4 for more details). The areas highlighted by gray shading in the left panels are depicted in detail in the right panels. All functionally important regions of the proteins that were identified by our method are shown in magenta with active site/substrate-binding residues in stick representation. Group 1: diagram from PDB entry 1df9 [43], a protease representing examples of fragments for which no PROSITE sequence patterns are available. The residues Pro132 and Gly133 make non-polar interactions with the residues of the NS3 protease (blue) inhibitor (cyan) at P2', while Ser135 and Ser163 make hydrogen bonds to side-chains of Ser21 at P1' and Lys20 at P1, respectively, of the inhibitor. Group 2: diagram from PDB entry 1e7u [44], representing examples for which PROSITE patterns are available but do not overlap with the fragments. The identified functionally relevant region is spatially contiguous to the PROSITE predicted residues; the critical Tyr867 residue implicated in ligand binding is highlighted as a stick model. Groups 3 and 4: diagram from PDB entry 1tgj [45], representing examples where PROSITE pattern overlaps with the fragment. The fragment derived sequence pattern overlaps with the amino-terminal part of the PROSITE pattern (PS00250), which is annotated as a cytokine involved in the repair of tissues. Trp30 and Trp32 interact with the bound dioxane.

No PROSITE sequence signature pattern is available for NS3 protease (PDB: 1df9A [43]). It was found that the first and third ranked fragments derived from level 3 GO propensity calculations encompass residues 132-141 and contribute residues to the binding pocket of the protease (Table 4). In particular, it has been shown [43] that Pro132 and Gly133 make van der Waals interactions with the P2' region of the Bowman-birk inhibitor while Ser135 and Ser163 participate in side-chain polar interactions with the inhibitor's polar atoms at Lys20 in the P1 site (Figure 4, Group 1). A fragment containing residue 163 (156-163) was found with a lower propensity value. It is interesting to note that residues 96-103, which represent fragments showing the second ranked propensity, form a scaffold for the active site, which corroborates its definite structural significance (*p*-values ≤0.05).

**Table 4:** Details of representative functionally important fragments of FL8 enumerated using GO level 3

PDB (group number)*	GO MF (EC number)	PROSITE pattern	Molecular function	Functionally important fragment(s) (start: sequence (propensity))^†^	*P*-value
1df9A (1)	0003724 (3.4.21.91)	-	Dengue virus NS3 protease	132: **PG**T**S**GSPI (30)	4.17e-5
				133: **G**T**S**GSPII (40)	5.95e-8
				156: TRSG**A**YV**S **(24)	0.007
1e7uA (2)	0016773 (2.7.1.153)	PS00915	Phosphatidyl-inositol 3- and 4-kinase signatures 1 and 2	857: TESLDLCL (48)	0.02
		PS00916		861: DLCLLP**Y**G (23)	0.04
				872: TGDKIGMI (29)	0.03
1tgj (3/4)	0005160	PS00250^‡^	Cytokines (repair of tissue)	27: DLG**W**K**W**VH (305)	0.04

*The chain identifier is given after the four letter PDB code, wherever present. ^†^Amino acids in bold either directly or indirectly participate in the enzyme function. ^‡^PROSITE pattern: (33-48, VHEPKGYYANFCSGPC).

#### Group 2: phosphatidylinositol kinase activity

In the protein (PDB: 1e7uA [44]) two PROSITE patterns (PS00915, residues 691-705, and PS00916, residues 790-810) describe the phosphatidylinositol 3-kinase and 4-kinase (EC 2.7.1.153) signatures 1 and 2 (Table 4), respectively. The top ranked fragment identified from our analysis (857: TESLDLCL) forms a rigid linker that contributes residues to the binding of ATP and/or inhibitors and are essentially in the binding pocket of the protein [44] (Figure 4, Group 2). On one end of this linker (872: TGDKIGMI), the backbone nitrogen of Val882 makes important hydrogen bonding contacts. Tyr867, which is part of two overlapping high propensity fragments (861: DLCLLP**Y**G), is critical to the binding of ATP and the inhibitor molecules. Experimental analyses show mutation at this position reduces lipid kinase activity to less than 10% of the wild-type enzyme. The integrity of the catalytic site is maintained by rigid packing around Tyr867, as evident from a mutation study in a phosphatidylinositol 3-kinase *γ *homolog, where a I963A modification completely abolished the catalytic activity [44].

#### Groups 3 and 4: growth factor β3

Growth factor β3 (PDB: 1tgj [45]) is described by a PROSITE pattern (PS00250) that corresponds to the transforming growth factor beta (TGF) family. The second ranked fragment identified at a level 3 propensity calculation starts at residue 27 and partly overlaps the PROSITE pattern (Table 4). The fragment contains two functionally critical residues. Trp30 and Trp32 interact with the dioxane, which has structural similarity to a carbohydrate moiety (Figure 4, Group 3). The Trp residues are shown to be involved in carbohydrate recognition [45]. It is noteworthy that the two Trp residues are totally conserved in the known TGF families, implying that these residues could be incorporated into the present PROSITE signature pattern, which would in turn enhance the functional prediction from the sequence. Other lower ranked overlapping fragments starting at residue 22 span the whole of the PROSITE pattern.

### Mapping high propensity fragments in proteins, and functional relevance

**Figure 5:**
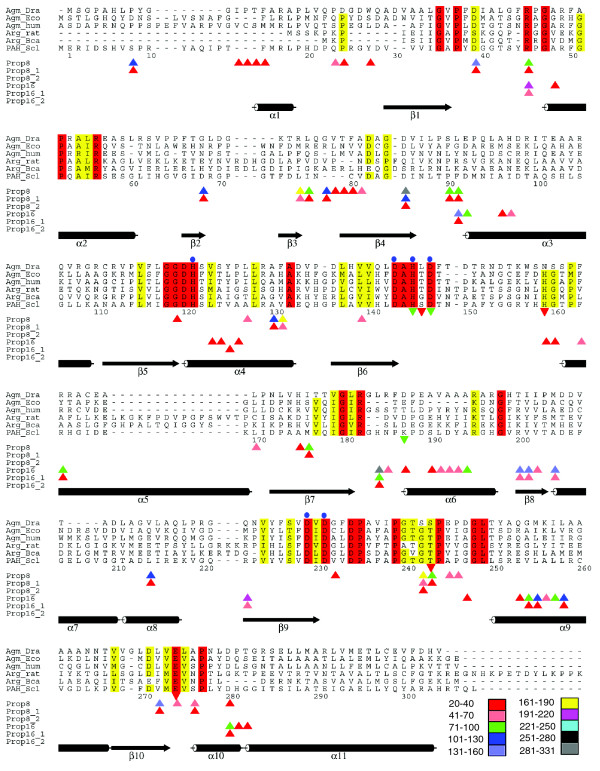
Mapping of high propensity fragments for PDB entry 1woh [30], shown on a backdrop of the multiple alignment of ureohydrolase superfamily enzymes. The start positions of high propensity fragments are marked by triangles in the last six rows of each panel. Binned propensity values are given in the color legend. Prop8, propensities derived from FL8, GO level 3 mapped from GO:0016813; Prop8_1, propensities derived from FL8, GO level 4 mapped from GO:0016813; Prop8_2, propensities derived from FL8, GO level 4 mapped from GO: GO:0046872; Prop16, Prop16_1, and Prop16_2 refer to the same information, except that it was derived from FL16. The residue numbers are indicated for 1woh, which is DR agmatinase: Agm_Dra (SWISS-PROT entry Q9RZ04). Other proteins in the alignment are Agm_Eco for agmatinase from *E. coli *(P60651); Agm_hum for agmatinase from human mitochondria (Q9BSE5, residues 1-35 deleted); Arg_rat for arginase I from rat liver (P07824); Arg_Bca for arginase from *Bacillus caldovelox *(P53608); and PAH_Scl for proclavaminate amidinohydrolase from *Streptomyces clavuligerus *(P37819). Secondary structure elements are shown as cylinders for helices and fat arrows for β-strands. Strictly conserved residues and semi-conserved residues are colored red and yellow, respectively. Above the sequences, blue circles indicate the residues that coordinate Mn^2+ ^ions. In the same panel as residue numbers, brick-red colored inverted triangles indicate residues putatively interacting with the guanidinium group of agmatine. Green inverted triangles indicate the residues observed in the crystal structure to be interacting with the bound inhibitor. Further details may be obtained from [30]. The figure was drawn using the program ALSCRIPT [69].

A protein can sometimes have many high propensity fragments and be annotated with multiple GO terms, giving rise to a peculiar situation while relating a fragment to its relevant GO MF term. In our calculations, since the propensity is derived after mapping the individual GO MF at a specific level from the fragment, the reverse mapping may not be unique. Therefore, although fragments may be of strong functional relevance as indicated by propensity calculations, they may not be uniquely identified with a specific MF. The possibility of specific mapping of fragments to relevant function increases as we perform our propensity calculations at deeper GO levels of 4 or more. As a case study we examined PDB entry 1woh [30], with only two GO terms, GO:0016813 and GO:0046872(Figure 2). PDB entry 1woh is a 305 residue agmatinase binuclear manganese metalloenzyme . The protein is without any PROSITE sequence pattern, yet a look at the propensity mappings showed some interesting trends (Figure 5). As can be seen from all propensity values ≥20 mapped to fragment start positions at different GO levels, large parts of the protein are covered by high propensity fragments, the coverage being more dense around conserved regions, especially around the functionally important residues. It may be noted that the fragments derived from the FL16 calculations occasionally overlap with the FL8 calculations at level 3. All fragments at level three are mapped through GO:0016813. But on using level 4 for propensity calculations, GO:00046872 could be mapped to only two functionally relevant fragments, one of which includes Ser243, which is a part of the active site. At level 5 no propensity calculations could be made for the protein because the deepest level of GO:0016813 and GO:0046872 is 4. Therefore, deeper level annotations are desirable for improved use of our methodology. It should also be noted that FL8 and FL16 results (shown as triangles in Figure 5) do not always necessarily overlap. Cases where they do not overlap occur where the FL8 fragment is completely contained in a regular secondary structure (like an *α*-helix), while the longer FL16 fragment starting around the same postion is long enough to extend beyond the same secondary structure segment (or *vice versa*). This causes the two fragments to have drastically different cluster populations in the final output, although they span the same protein segment, resulting in significantly different GO propensities. It appears that propensity values from longer FLs in such cases should be cautiously interpreted to make a combined evaluation. These observations indicate that the best assessment of functional relevance of the fragments through GO-based propensity is dependent on both the optimal length of the fragment chosen for clustering as well as the level of the GO MF used for the calculation. A systematic study to delineate these issues is underway.

### Features of high propensity (≥20) fragments

**Figure 6:**
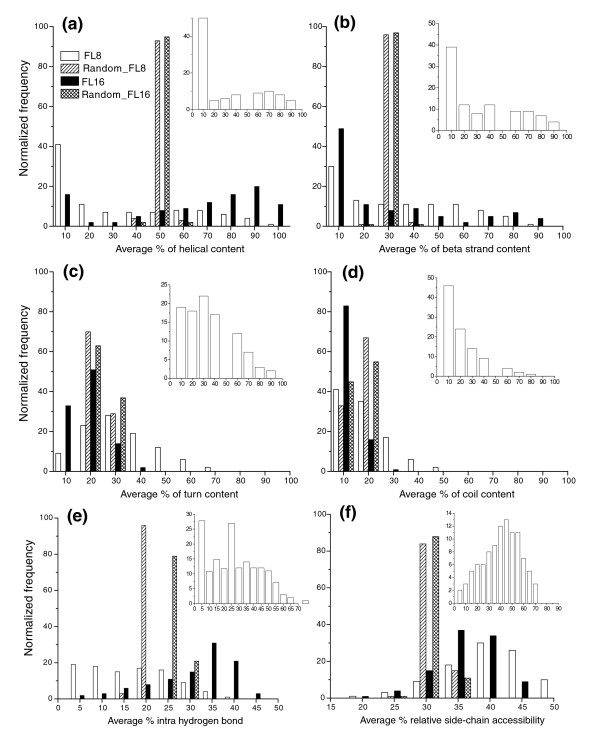
The distribution of secondary structural content in observed and pseudo-clusters of FL8 and FL16. The statistical significance of the observed distribution can be estimated by comparing the respective plots for the pseudo-clusters. **(a) **helical; **(b) **β-strand; **(c) **turn; **(d) **irregular secondary structure. **(e,f) **Plots of normalized frequency of average percent of intra-hydrogen bonds (e), and percent relative side chain accessibility (f). The x- and y-axes of insets are the same as in the main figures, and depict information from the functionally important fragments with propensity ≥20 identified in this work.

There are 4,400 (from 526 PDB entries) 8-mers with propensity ≥20. For these fragments, since we know that a majority are directly related to protein biochemical function, we sought to ask if they had any unique features in terms of distribution of secondary structure, hydrogen bonding, surface accessibility and hydrophobic content preferences (Figure 6, insets). The overall distribution of secondary structures and hydrophobicity properties was found to be similar with respect to the distribution observed for the entire clustered data set (Figure 6, main plots). Substantial differences were noticed for the hydrogen bonding pattern and relative side-chain accessibility. A considerable number of functional fragments are stabilized by inter-fragment hydrogen bonds and more than 50% of them have a relative side-chain surface accessibility of greater than 30. This may be due to the fact that functional residues are positioned strategically and often they are surface exposed. Below we describe cluster properties in more detail.

### Secondary structure content

The percentages of secondary structures (H = helical, B = beta, T = loop, C = irregular structure) of residues in all functionally important FL8 fragments (propensity ≥20) identified in this work are plotted in the inserts of Figure 6a-d. The same plot was drawn taking average secondary structure content in a cluster. We found that the distributions of the secondary structures in both sets are approximately similar; only for turns is the peak in the 0-10% content range increased four-fold compared to the corresponding peak for all FL8 clusters. Looking at the general features of the clusters, we find that the FL8 clusters have lower helical content than FL16 clusters. The fraction of clusters having minimal (0-10%) helical content decreases more than half from 43% to 17% for FL8 and FL16, respectively. The trend is reversed for β-strands, where it is known that the mean length is between five and six residues [46]. The content of both turns and irregular secondary structure in clusters is significantly restricted between 0% and 30%. More importantly, these distributions are similar to those from randomly shuffled pseudo-clusters, suggesting that turns and coils have a minor role in cluster formation based on conformation. There are only a few turn and coil dominated functional fragments. It may be noted that the distribution of helical and β secondary structures from randomly shuffled pseudo-clusters is more narrow in contrast to observed clusters, suggesting that precise combinations of secondary structural elements are essential for formation of structural motifs. This is consistent with the fact that permutations of secondary structural elements result in divergence and new topologies [47].

### Hydrogen bonding

We calculated the ratio of intra-fragment hydrogen bonds to all the hydrogen-bonding contacts made by the individual fragment. Looking at the distribution of intra-fragment hydrogen bonding in functionally important fragments (Figure 6e, inset) suggests that availability of unsatisfied hydrogen bonding potential of fragments is important for function, as manifested by low occurrence of intra-fragment hydrogen bonds (higher peak in 0-5 range). Looking at the average fraction of intra-fragment hydrogen bonds in clusters, the number of clusters with no intra-molecular hydrogen bonds is highest for FL8; the trend is reversed for FL16, where helical content is significantly higher (Figure 6a). As can be seen, the major peak for FL8 at 20% is shifted to 25% in FL16 in pseudo-clusters; this suggests that among other intermolecular interactions, the ubiquitous presence of hydrogen bonding is the major driving force for large or supersecondary structural motif formations in proteins.

### Relative side-chain accessibility

Functional residues have a distinct preference for either full burial or high solvent exposure; as a result the plot for the solvent exposure (Figure 6f) has two peaks, one at 0-25 Å^2 ^and another at 30-70 Å^2^. This is in contrast to the unimodal distribution of average solvent exposure of clusters centered at 30-40 Å^2 ^for both FL8 and FL16. The same calculations using pseudo-clusters show a peak at a greater burial than the mean of the FL8 and FL16 observed distribution, suggesting that structural motifs do prefer more exposed locations in the tertiary structure, in contrast to both buried and exposed functional motifs.

### Hydrophobic content

All fragments, including functionally important ones, show a non-preferential hydrophobicity distribution. We calculated hydrophobicities of functionally important fragments and the average hydrophobicites of clusters using Wolfenden [48] and Kyte-Doolittle [49] scales. The graphs show normal distributions for both the scales, as well as with calculations using pseudo-clusters; all graphs for a given scale share the major peak around the same bin (data not shown).

### Conformational diversity of identical sequences and implications for protein function

The presence of identical peptide fragments in multiple clusters offers lessons for protein engineering, design and functional requirement/perturbation arising from conformational promiscuity. It has been previously shown that identical peptides can have completely different conformations in unrelated proteins [50,51]. We revisited the previous observation by analyzing our clustering results, including the data set from FL5. The clustering of penta-peptide fragments showed nearly 10.4% (0.16% for the FL8 data set) of the fragments in the clusters (47,227 out of 455,305) to have at least two different conformations (Table 5). Further, the nature of structural transition between the conformations was analyzed using secondary structure definition according to the DSSP algorithm [52]. Only four different secondary structural states (H, B, T and C) were considered for a residue in a fragment. For each identical sequence found in more than one cluster, the conformational state at each position of the fragment was matched/compared to identify the structural transition between them. It is noteworthy that 42% of the FL5 repeat sequences have no match in all of the five-positions, implying they are totally dissimilar conformations (Table 5). When the analysis was repeated using FL8 fragments, the fraction decreased to 4.6%, while at FL16, no identical fragments were found across multiple clusters. Looking at identical sequences found across multiple clusters, 10.2% of the FL5 sequences are found across 2 clusters; whereas only 1.5% of sequences are found across 3 or more clusters. The sequence SGPSS, an all *trans *peptide, was found across a maximum of 32 clusters. Interestingly, when an identical sequence is found across more clusters, the difference in secondary structure tends to become less; as a result, there are only limited variations in the actual three-dimensional conformation of the fragments.

**Table 5:** Statistics on identical sequences occurring across clusters

Number of times found across the clusters	Number of sequences (percentage)		Number of matches between the conformational states	Number of cases (percentage)	
			
	FL5	FL8		FL5	FL8
1	41,716 (88.3)	693 (98.4)	0	22,875 (41.8)	33 (4.6)
2	4,819 (10.2)	10 (1.4)	1	8,181 (15.0)	42 (5.9)
3	528 (1.1)	1 (0.2)	2	7,104 (13.0)	54 (7.5)
4	69 (0.2)		3	6,484 (11.8)	72 (10.1)
5-32	11-1 (0.2)		4	5,505 (10.1)	77 (10.8)
			5	4,542 (8.3)	94 (13.1)
			6		128 (17.9)
			7		101 (14.1)
			8		115 (16.1)

We also checked which sequentially identical FL8 fragments present across multiple clusters had a high propensity. We found 235 (some of them overlapping) fragments from 57 different PDB files with propensity ≥5 and *p*-value ≤0.05. Of these, only 93 sequences from 31 PDB files had propensity ≥20.0. We randomly selected a few of these to assess how these conformationally promiscuous fragments were functionally relevant to the protein activity (Table 6). We found five sequences from the amino-terminal extracellular domain intradiskal loop of rhodopsin (PDB: 1u19A [53], 1edsA [54], 1edxA [54], 1edvA [54]) potentially involved in G-coupled signaling activity; the importance of conformational transition in G-coupled signal transduction is fairly well studied. In the eukaryotic translation initiation factor (PDB: 1kl9 [55]), the intra- and inter-domain movements are critical for tRNA binding during translation. Interestingly, our method revealed a fragment from human transforming growth factor β3 (PDB: 1tgj [45]) containing cysteine residues that were found to destabilize the protein when the disulfide bond was reduced. This hints at the important role of the fragment in conformational stability of structure and function. In PDB entry 1q9b [56], a IgE-binding natural allergen, the predicted fragments spanning residue positions 6-22 form the part of the conformational epitope experimentally observed to impart binding activity through Trp. In the P-type ATPase family, Ca^+2^-ATPase of the skeletal muscle sarcoplasmic reticulum contains a flexible fragment experimentally corroborated and also found in this study (PDB: 1wpgA [57]). This fragment spanning residues 349-357 contains an Asp at position 351 that is phosphorylated, triggering this conformational transition. A similar example from *Neurospora *plasma membrane H^+ ^ATPase, spanning fragment 377-384 found in this study, contains an Asp at position 378 that is reversibly phosphorylated, which triggers a conformational change in the protein, allowing it to function as a proton pump (PDB: 1mhsA [58]). Interestingly, additional conformationally flexible fragments spanning 631-640 revealed by this study lie in a spatially contiguous location to fragment 377-384, indicating the requirement of conformational flexibility of not only the fragment triggering the transition, but also the neighboring segments. These results highlight how our propensity-based method is able to screen for functionally important fragments, selecting protein segments influencing dynamic structure and plasticity.

**Table 6:** Identical sequences of FL8 present across multiple clusters with GO MF propensity calculated using level 3*

PDB [reference]^†^	Molecule	Putative fragment function	Sequence (propensity)§	*P*-value
1u19A^‡^ [53]	Rhodopsin	Part of extracellular domain intradiskal loop involved in cell signaling	11: VPFSNKTG (47)	0.02
1edsA [54]	Bovine rhodopsin	Same as above	17: GCNLEGFF (93)	0.01
			21: EGFFATLG (39)	0.03
			22: GFFATLGG (130)	0.008
1edvA [54]	Bovine rhodopsin	Same as above	16: CGIDYYTPP (96)	0.01
1edxA [54]	Bovine rhodopsin	Same as above	11: VPFSNKTG (22)	0.04
1tgj^‡^ [45]	Human transforming growth factor β3	Structure destabilized on dislufide bond reduction	72: ASASPCCV (157)	0.006
1kl9A^‡^ [55]	Human translation initiation factor 2α	Linker for the penultimate 3_10_ helix and the last α-helix in domain 1	163: DSLDLNED (35)	0.03
			164: SLDLNEDE (35)	0.003
1q9bA^‡^ [56]	Hevein (IgE bonding natural allergen)	Part of conformational epitope	6: QAGGKLCP (62)	1.3e-08
			8: GGKLCPNN (299)	2.3e-08
			9: GGLCPNNL (123)	9.8e-12
			11: LCPNNLCC (25)	1.3e-06
			12: CPNNLCCS (28)	2.0e-08
			14: NNLCCSQW (28)	≈ 0
			15: NLCCSQWG (79)	1.5e-08
1wpgA^‡^ [57]	Sarcoplasmic/endoplasmic reticulum calcium ATPase	Phosphorylation of D351 causes the protein to switch conformation	349: CSDKTGTL (41)	0.002
			350: SDKTGTLT (56)	0.001
1mhsA^‡^ [58]	Proton ATPase	Phosphorylation of D378 causes the protein to switch conformation	631: MTGDGVND (22)	0.008
			633: GDGVNDAP (25)	0.04
			376: CSDKTGTL (41)	0.002
			377: SDKTGTLT (56)	0.001

*The highest propensity fragment from only one cluster is shown. ^†^Files indicated in regular font denote an NMR-derived structure. ^‡^An X-ray-derived structure. The chain identifier is indicated after the four letter PDB code, wherever present. ^§^Disulfide bonded Cys are underlined.

## Discussion

Clustering peptide fragments has been long practiced by structural biologists as a means to understand protein features; however, our method of assessing fragment-function links using GO has not been done before. The existing approaches of function assessment mostly use information at some level from either annotated sequence or structure information for prediction/mapping of the functional regions in protein structures (for example, Espadaler *et al*. [21]). In contrast, our method does not use prior knowledge on fragments; most importantly, only GO terms and a group of geometrically similar fragments are considered for dissecting the functional regions. The procedure we follow consists of three steps. In the first step we cluster the fragments based solely on geometric considerations using backbone torsion angles. This identifies a conformationally similar set of peptides. It is important to note that at this stage of the grouping, fragments from all parts of the protein structure, not solely those restricted to loops and turns, are taken into account. In the second step, we assign molecular functions to the fragments in a given cluster from level-specific mapping of molecular function terms using the GO graph. In the third step, we identify statistically significant benchmarks for protein fragments that are reliably associated with MF. This novel composite procedure has helped in delineating new protein fragments associated with function. Another attractive feature of our method is that we characterize functions of fragments at different levels of the GO, which allows for continual improvement as the GO database grows.

The method of agglomerative clustering as implemented is also new as applied to the protein fragments. Our method is unique because of the self-organizing ability of the cluster centers; this allows the clusters to be centered on the densest distribution of points in the torsion space. Moreover, we use two distance measures to group the fragments: the first is the Euclidian distance between the φ,ψ torsion angles of the fragment and the cluster center, and the second is the pair difference between torsion angles at equivalent positions of the fragment under consideration and the cluster center. While the former gives a global measure of similarity, the latter indicates the local similarity. The two distances in combination give a conformationally homogenous distribution of fragments in the cluster in a way that facilitates their dissection according to functional importance.

It is not our claim that our method is computationally superior to or computationally more efficient than other methods assessing function. We would like to emphasize that ours is an entirely new method that enables discovery of new sets of fragments associated with function in a statistically rigorous fashion. It can be alluded to as a protein-fragment-geometry derived assessment method, where instead of using primary sequence information to derive function from canonical sequence-structure-function relationships, we have used geometric alignment and the GO to dissect important fragments linked to function. While structural comparison works well at the level of protein fold, at smaller structural sizes many diverse sequences may have similar conformations, making difficult the decomposition of fragment functional properties in a quantitative way. Our propensity calculations are able to filter a subset of fragments that may indeed be linked to the protein function. *P*-values calculated using the hypergeometric distribution lend credence to the results in a statistically rigorous fashion.

The utility of the method to the biologist is multifarious. For example, once a fragment has been identified that can be linked to function, this information is useful for assessing putative functions of new proteins, as well as guiding protein engineering experiments or designs with desired functionalities. Our example of PDB entry 1woh [30] shows how fragments proposed from our method map on to functionally important and sequentially conserved regions of the molecule. It also raises an important question as to whether our method can predict important fragments for all proteins, since every protein has a function. In principle, this is possible as we can extend the coverage of our method by varying the clustering parameters, and make it more selective by sub-clustering to better assess the ranking/importance of fragments *vis-à-vis *their direct relevance to MF. A fragment library created from such high propensity fragments can be used in annotating proteins with unknown function. In these cases the calculations are preferably done at a deeper level of 5 or more in the GO directed acyclic graph, and appropriate propensity value thresholds should be used for screening the fragments after plotting the propensity distribution.

Proteins containing high-propensity fragments as identified by our methodology appear to be ideal candidates for protein engineering and design experiments, as they provide functionally important sites that can be targeted for inhibition. As can be seen, the ranges of functions in which the fragments are important include both enzymatic and non-enzymatic functions. For example, in PDB entry 1df9 [43], which is a Dengue virus protease that processes polyproteins, residues that interact with the substrate (Asp129, Tyr150 and Ser163) are absolutely conserved among almost all of more than 70 flaviviruses. But our conformational analysis suggests that fragments spanning residues 132-140, and 156-163 are also very important in providing the correct receptor site for the substrate. Therefore, mutation in these regions would also modulate the turnover of the protease as well as its specificity for substrate.

While making decisions on protein design one can make useful inferences from our clustering results based on variation of structural stability with peptide lengths. Similarly, sequences that are conformationally promiscuous can be easily recognized and included/excluded during design as needed. Coupling protein fragments with function using propensity also provides a useful opportunity for understanding the amyloidogenic propensity of peptides [59] and drug targets, especially in 'conformational diseases'.

Although secondary to the main objectives of this work, the clustering results obtained are of direct interest in understanding the inverse protein-folding problem. Of the FL8 fragments, 92% have a partner with similar conformation. This suggests that efficient assembly of protein folds based on fragments is realistically possible. Two important observations available from Figure 6 are the role of hydrogen bonds in accommodating a given conformation, and the importance of the order of secondary structures in the polypeptide chain, rather than the overall hydrophobicity in accommodating diverse sequences into a specific fold. It may be noted that the data set we have chosen is highly unbiased, because each protein in the data set is a distinct fold. The amino acid identity between proteins is therefore expected to be below 20%. Therefore, our data reflect which unbiased properties may be essential in making diverse sequences compatible to a given fold. Further property-based sub-clustering will be useful in these regards for development of *ab initio *methods of protein modeling.

## Conclusion

Our proposed clustering-cum-function analysis method is useful in dissecting/identifying protein fragments based on their relevance to function. Its application to propensity-based functional inference on identical fragments across multiple clusters highlights its diverse utility. In particular, the absence of any sequence alignment step in the method makes it a valuable tool to predict functionally important regions in hypothetical proteins from structural genomics projects. The data provided by the method comprise a nucleus on which our future sequence-cum-geometric signature pattern libraries will be developed. It will benefit function annotation efforts, as well as protein engineering, design and modeling studies.

## Materials and methods

### PDB files

The list of PDB files for clustering was obtained from the DALI Domain Dictionary [60] by choosing one representative PDB entry per fold (Additional data file 1). The PDB file with best resolution and R-factor was chosen.

### Secondary structure representation

The backbone torsion angles of each PDB file were assigned using the program SECSTR of the PROCHECK suite [61]. The secondary structure of each residue was classified into four states, helical (H), β-strand (B), loop (T) and irregular structures (C) for each residue in a fragment. Symbols H/h, G/g, and P/p denoting α-helix, 3_10_-helix, and π-helix, respectively, were merged and treated as H; E/e and B, denoting β-strand and β-ladder, respectively, were merged and treated as B; T/t and S/s, denoting turn and geometrical bends, respectively, were merged and treated as T; blank, denoting irregular secondary structure, were treated as C.

### Clustering procedure

To cluster the fragments from a protein structure, the backbone is divided serially into overlapping fragments with specified FL and torsion (φ,ψ) angles for the fragment residues and put into an array. Because the terminal residues (or where there is a chain break) of the protein do not have φ/ψ angles, these residues are not included in the fragment. Also, residues with main-chain atoms with a B-factor >60 Å^2 ^are rejected. This ensures that in the absence of a threshold resolution and R-factor for selecting structures modeled from electron densities, we chose fragments that did not incorporate large coordinate errors. For NMR derived structures, we always chose the first model in the PDB file. The omega angles were checked to ensure all the peptide bonds are *trans *in the fragment. Any fragment with a *cis *peptide bond was ignored for our current analysis. A peptide bond is considered to be a *cis *bond if the absolute value of the omega angles are less than or equal to 90°. For a fragment length of 8, eight pairs of dihedral angles will be used for clustering (FL = 8).

For each protein of length n to be included in the search, we first compute the following series of dihedral angles: {(*φ*,*ψ*)_1 _(*φ*,*ψ*)_2 _(*φ*,*ψ*)_3 _(*φ*,*ψ*)_4 _(*φ*,*ψ*)_5 _(*φ*,*ψ*)_6 _(*φ*,*ψ*)_7 _(*φ*,*ψ*)_8 _(*φ*,*ψ*)_9 _(*φ*,*ψ*)_10 _(*φ*,*ψ*)_11 _(*φ*,*ψ*)_12 _... (*φ*,*ψ*)_n-1_ (*φ*,*ψ*)_n_}, where *n *is the number of amino acids used to obtain the fragments from a protein structure. The peptide chain is then decomposed into a series of overlapping fragments of specified length (FL = 8, for example, as depicted below):

*F*_1_: [(*φ*,*ψ*)_2 _(*φ*,*ψ*)_3 _(*φ*,*ψ*)_4 _(*φ*,*ψ*)_5 _(*φ*,*ψ*)_6 _(*φ*,*ψ*)_7 _(*φ*,*ψ*)_8 _(*φ*,*ψ*)_9_]

*F*_2_: [(*φ*,*ψ*)_3 _(*φ*,*ψ*)_4 _(*φ*,*ψ*)_5 _(*φ*,*ψ*)_6 _(*φ*,*ψ*)_7 _(*φ*,*ψ*)_8 _(*φ*,*ψ*)_9 _(*φ*,*ψ*)_10_]

*F*_*n*-7_: [(*φ*,*ψ*)_n-8 _(*φ*,*ψ*)_n-7 _(*φ*,*ψ*)_n-6 _(*φ*,*ψ*)_n-5 _(*φ*,*ψ*)_n-4 _(*φ*,*ψ*)_n-3 _(*φ*,*ψ*)_n-2 _(*φ*,*ψ*)_n-1_]

We define the distance between two fragments [*F*_*i*_, *F*_*j*_] as:

$$DIST_{\left[Fi,Fj\right]}={\left[\sum_{x=l,y=m}^{l+7,m+7}{\left(\varphi_{ix}-\varphi_{jy}\right)}^{2}+\sum_{x=l,y=m}^{l+7,m+7}{\left(\psi_{ix}-\psi_{jy}\right)}^{2}\right]}^{1/2}$$

where *l, m *are the starting positions of the fragments [*Fi, Fj*], respectively.

For every (*ψ*_*im*_-*ψ*_*jm*_), **if **|*ψ*_*im*_-*ψ*_*jm*_| > 180,

**then **use 360 - |*ψ*_*im*_-*ψ*_*jm*_|

For every (*φ*_*im*_, *φ*_*jm*_)) **if **|*φ*_*im*_-*φ*_*jm*_| > 180,

**then **use 360 - |*φ*_*im*_-*φ*_*jm*_|

Assume a set of similar fragments forms a group and *L *is the index label that identifies the groups. We define the center of group *L*, *C*_*L*_, as [(*φ*_*j*1_, *ψ*_*j*1_), (*φ*_*j*2_, *ψ*_*j*2_), ... (*φ*_*j*8_, *ψ*_*j*8_)], where:

$$\begin{array}{cc} \varphi_{jm}=(\sum_{i=1}^{N_{L}}\varphi_{im})/N_{L};\psi_{jm}=(\sum_{i=1}^{N_{L}}\psi_{im})/N_{L}, & \left(\text{m}=1,2,...8\right) \end{array}$$

where *N*_*L *_is the number of fragments *F *in the group, and the sum is over *i*. The cyclic nature of the (*φ*,*ψ*) values has been preserved by adding -360° if any *φ*/*ψ *is >180° or by adding 360° if any *φ*/*ψ *is <-180°. The distance between fragment *F*_*i *_and the center of group *L*, *C*_*L *_is given as ***DIST***_*[Fi, CL]*_.

### Algorithm

**Input: **a set of *φ*,*ψ *from *F*

**Output: **a set of groups into which the points have been divided, where every point in a group is within the distance (***DIST***) threshold ***R ***from its group center *C*_*L *_and angle difference at each position in the fragment and group center *C*_*L *_does not exceed ***ANG***.

Begin

**I. **Pick an arbitrary fragment (it is the seed fragment and starting cluster center C_1_)

Until the last remaining fragment do

{

Find distances between *C*_*L *_(*L *= 1, *L*_*max*_) and the fragment *F*_*k*_.

*L*_*max *_= maximum number of cluster centers existing at that point of time.

*φ*_*iCL*_-*φ*_*iFK *_= *φ *angle difference at position *i *in cluster center L and fragment K.

*ψ*_*iCL*_-*ψ*_*iFK *_= *ψ *angle difference at position *i *in cluster center L and fragment K.

**If *DIST***_[*CL*, *Fk*] _≤ ***R ***and (*φ*_*iCL*_-*φ*_*jFK*_) ≤ ***ANG ***and (*ψ*_*iCL*_-*ψ*_*jFK*_) ≤ ***ANG***{

Insert *F*_*k *_into group *L *and add 1 to *N*_*L*_

Compute the new center *C*_*L*_' of group *L*

} **Else **{make the fragment a new cluster center *C*_*L*+*1*_}

}

**II. **For each fragment in the list {

a). Find distances between *C*_*L *_(*L *= 1, *L*_*max*_) and the fragment *F*_*k*_.

**If *DIST***_[*CL*, *Fk*] _> ***R ***or (*φ*_*iCL*_-*φ*_*jFK*_) > ***ANG ***or (*ψ*_*iCL*_-*ψ*_*jFK*_) > ***ANG***     {

1. Reject *F*_*k *_from group *L *and subtract 1 from *N*_*L*_

2. Compute the new center *C*_*L*_' of group *L*

3. Do a). for fragment *F*_*k*_.

**If *DIST***_[*CL*, *Fk*] _≤ ***R ***and (*φ*_*iCL*_-*φ*_*jFK*_) ≤ ***ANG ***and (*ψ*_*iCL*_-*ψ*_*jFK*_) ≤

*ANG*

{

Insert *F*_*k *_into group *L *and add 1 to *N*_*L*_

Compute the new center *C*_*L*_' of group *L*

} **Else **{make the fragment a new cluster center *C*_*L*+*1*_}

}

b). Keep count of number of fragments rejected

}

If number of fragments rejected in previous round > current round do { **II **}

else { print cluster details}

END

For our clustering runs, we used ***R ***= 30° × X, where X is the fragment length and ***ANG ***= 60°. The code has been implemented in PERL and is available from the authors upon request.

### Generation of pseudo-clusters

Clusters are built by randomly picking fragments from the total fragment library of a given length. The total number of fragments in each set of pseudo-clusters added up to 100,000 fragments. The distribution of physicochemical properties of clusters was averaged over 30 generated sets in order to generate base values for the estimate of statistical significance.

### Identification of functionally important fragments

The GO term, which corresponds to the MF of the protein in the PDB, was taken from the GOA annotation [62]. Accordingly, each fragment in the cluster was assigned to a GO MF term of its PDB entry. The parent functions for each fragment MF term at a given level from the root node were identified from the GO directed acyclic graph (Figure 2). We have carried out the analysis at levels 3, 4, and 5 (level 3 implies that the parent is at three edges from the root node GO:0003674). The propensity was calculated for each fragment function in a cluster using the following formula:

PropensityL=(nXLnTL)/(NXLNTL)

where *n*_*X *_and *N*_*X*_ are the number of GO MF term 'X' in a cluster and in all clusters, respectively, and *n*_*T *_and *N*_*T *_stand for the number of all functions in that particular cluster and in all clusters, respectively. *L *stands for the GO level at which the MF was mapped for the calculations. CATH identifier based propensity calculations were done the same way by replacing the GO term, wherever the CATH identifier for a protein was available. *P*-values for individual GO terms were calculated using the hypergeometric distribution formula as follows:

$$H_{L}(n_{XL};N_{TL},n_{TL},N_{XL})=\frac{\left(\begin{array}{l} n_{TL}\\ n_{XL} \end{array}\right)\left(\begin{array}{l} N_{TL}-n_{TL}\\ N_{XL}-n_{XL} \end{array}\right)}{\left(\begin{array}{l} N_{TL}\\ N_{XL} \end{array}\right)}$$

where symbols are the same as in the propensity equation. The probability of a GO term X among *K *GO terms in a cluster is given by $1-\sum_{t=0}^{K-1}H(t)$, and applying the Bonferroni correction, the *p*-value of the GO term X occurring *k *times in the cluster is $k\times (1-\sum_{t=0}^{K-1}H(t))$. A canonical threshold of ≤0.05 was used to identify the statistically significant fragments using the said formula.

For the structure-sequence pattern analysis, each sequence of all the fragments with propensity ≥20 was searched with the program BLAST [63] using short and nearly exact match against the UNIPROT database [64] of sequences. The hits with at least one PDB entry were taken for further PROSITE pattern searches. The full sequences of such fragments with one PDB hit were scanned for PROSITE sequence signature patterns and subsequently classified into different groups (see Results for details). The selection scheme was used to filter down the number of possible hits to be manually reviewed from the literature, and also test if the fragments alone are able to pick out homologous PDB sequences, which could be further used for detailed investigations as needed.

### Information content

The information content of the fragments was obtained using the Shannon entropy measure formula [65]. For a given position in the fragment, the entropy was calculated as:

*S *(at a given position) = -∑*w *log(*w*)

where the summation runs over all amino acids and *w *stands for the fraction of occurrence of each residue at that position. An average of entropies at each position was taken to calculate the average information content of the cluster. A value *S *= 0 means that the position is fully conserved and a more positive *S *implies the position is diverse in amino acids.

### Surface accessibility

The percent relative side-chain accessibility of the fragments in a cluster was calculated using the program NACCESS [66] with a probe radius of 1.4 Å. A standard Ala-X-Ala tripeptide in extended conformation was used for calculation of percent relative accessibility.

### Hydrogen bonds

Hydrogen bonds were calculated using HBPLUS [67] with hydrogen bonding parameters (D-A distance ≥ 3.9 Å, H...A ≥ 2.7 Å, D-H...A ≥ 90°).

## Abbreviations

B, beta; C, irregular structure; FL, fragment length; GO, Gene Ontology; H, helical; MF, molecular function; PDB, Protein Data Bank; T, loop; TGF, transforming growth factor.

## Authors' contributions

KM wrote programs, carried out analysis and provided help with the literature review and drafting of the manuscript. DP designed and conceived the study, wrote programs, performed analysis and drafted the manuscript. SR participated in conceiving the study, provided input into the design of the study and helped in reviewing the manuscript drafts. NEB, SSI, and GS participated in mathematical formulation of the clustering algorithm. All authors read and approved the final manuscript.

## Additional data files

The following additional data are available. Additional data file 1 is a table listing the PDB files used in this work, culled from the FSSP library. Additional data file 2 is a histogram showing the distribution of compactness values for FL8 and FL16 clusters.

## Supplementary Material

Additional data file 1 PDB files used in this work, culled from the FSSP library. Click here for file

Additional data file 2 Distribution of compactness values for FL8 and FL16 clusters. Click here for file
