# Supplementary material for: Functionally important segments in proteins dissected using Gene Ontology and geometric clustering of peptide fragments
Source: Genome Biol. 2008 Mar 10;9(3):R52. doi: 10.1186/gb-2008-9-3-r52 (PMC2397504; doi:10.1186/gb-2008-9-3-r52)
Supplement: Additional data file 1 — PDB files used in this work, culled from the FSSP library. [file gb-2008-9-3-r52-S1.pdf]

Table S1. List of PDB files used in our work culled from FSSP library

PDB Code	Chain Identifier	Number of residues considered*	Resolution (Å^2)	R-factor
1a0a	A	63	2.8	0.23
1a0i	A	315	2.6	0.222
1a0r	P	188	2.8	0.228
1a1t	A	55	N/A	
1a26	A	335	2.25	0.168
1a2z	A	220	1.73	0.18
1a34	A	143	1.81	0.179
1a3a	C	146	1.8	0.19
1a3k	A	137	2.1	0.17
1a3q	A	250	2.1	0.219
1a5j	A	110	N/A	
1a6f	A	106	2.6	0.205
1a6q	A	359	2	0.214
1a6r	A	458	2.05	0.193
1a6s	A	87	N/A	
1a76	A	315	2	0.214
1a79	A	166	2.28	0.2
1a87	A	286	3.1	0.173
1a9o	A	283	2	0.2
1aa0	A	112	2.2	0.216
1aa3	A	63	N/A	
1aa7	A	158	2.08	0.208
1abe	A	302	1.7	0.137
1abv	A	105	N/A	
1aci	A	76	N/A	
1acz	A	108	N/A	
1adq	H	120	3.15	0.225
1ady	A	382	2.8	0.26
1ae9	A	171	1.9	0.199
1af7	A	274	2	0.2
1afo	A	40	N/A	
1afp	A	51	N/A	
1agq	B	96	1.9	0.203
1ah1	A	129	N/A	
1aho	A	62	0.96	0.158
1aj6	A	193	2.3	0.195
1ajj	A	37	1.7	0.209
1ak0	A	264	1.8	0.207
1ak2	A	214	1.92	0.222
1akl	A	470	2	0.197
1ako	A	268	1.7	0.169
1am7	A	149	2.3	0.163
1amx	A	150	2	0.2
1anv	A	297	2.7	0.204
1ao7	E	168	2.6	0.245
1aoc	A	146	2	0.191

1aoh	B	147	1.7	0.194
1aoj	A	60	2.5	0.193
1aol	A	228	2	0.223
1aov	A	611	4	0.21
1aox	A	196	2.1	0.219
1aoy	A	78	N/A	
1ash	A	147	2.15	0.179
1at0	A	142	1.9	0.218
1atg	A	231	1.2	0.164
1au7	A	130	2.3	0.23
1aun	A	208	1.8	0.169
1awd	A	94	1.4	0.146
1awj	A	77	N/A	
1ay2	A	158	2.6	0.194
1azo	A	211	1.7	0.203
1azs	A	112	2.3	0.219
1azt	B	333	2.3	0.219
1b0n	A	103	1.9	0.199
1b0u	A	258	1.5	0.185
1b12	A	235	1.95	0.22
1b22	A	70	N/A	
1b25	A	611	1.85	0.174
1b2m	A	104	2	0.187
1b2p	A	119	1.7	0.186
1b2u	D	90	2.1	0.214
1b2v	A	173	1.9	0.175
1b35	B/D	246/57	2.4	0.228
1b3q	B	243	2.6	0.213
1b3u	A	568	2.3	0.209
1b4a	A	122	2.5	0.224
1b4u	A/D	132/295	2.2	0.161
1b5s	A	242	4.4	N/A
1b63	A	328	1.9	0.213
1b65	A	363	1.82	0.169
1b67	A	68	1.48	0.188
1b6z	A	138	2	0.216
1b8i	A	58	2.4	0.224
1b9n	A	242	2.09	0.214
1b9o	A	123	1.15	0.122
1b9w	A	87	1.8	0.212
1bal	A	51	N/A	
1baw	A	102	2.8	0.193
1bb1	A	34	1.8	0.208
1bbg	A	40	N/A	
1bbh	A	131	1.8	0.185
1bbp	A	173	2	0.2
1bbu	A	375	2.7	0.228
1bc2	A	208	1.9	0.208
1bcm	A	304	2.8	0.214
1bcp	D	110	2.7	0.232
1bd2	D	159	2.5	0.238
1bd8	A	156	1.8	0.19

1bdf	B	227	2.5	0.225
1bds	A	43	N/A	
1bed	A	171	2	0.205
1bei	A	34	N/A	
1bev	4	17	3	0.225
1bg1	A	331	2.25	0.246
1bg6	A	349	1.8	0.188
1bgc	A	157	1.7	0.213
1bgf	A	124	1.45	0.188
1bgk	A	37	N/A	
1bgp	A	308	1.9	0.192
1bgw	A	669	2.7	0.22
1bh7	A	31	N/A	
1bhg	A	611	2.53	0.231
1bht	A	171	2	0.196
1bhu	A	102	N/A	
1bi6	H	41	N/A	
1bi9	D	408	2.7	0.234
1bkb	A	132	1.75	0.214
1bkr	A	108	1.1	0.141
1ble	A	88	2.9	0.185
1blu	A	80	2.1	0.192
1bm4	A	32	N/A	
1bml	D	202	2.9	0.201
1bmw	A	94	N/A	
1bnz	A	64	2	0.168
1bo1	A	246	3	0.229
1bob	A	266	2.3	0.204
1bor	A	56	N/A	
1bpl	A	179	2.2	0.167
1bq9	A	53	1.2	0.137
1bqs	A	176	2.2	0.223
1bqu	B	215	2	0.221
1bqv	A	110	N/A	
1bs3	A	201	1.55	0.174
1bte	B	94	1.5	0.181
1bu8	A	446	1.8	0.202
1bus	A	56	N/A	
1bv8	A	138	N/A	
1bvs	A	140	3	0.274
1bw9	A	349	1.5	0.195
1bwx	A	39	N/A	
1bx4	A	342	1.5	0.192
1bx7	A	51	1.2	0.179
1bxe	A	108	1.9	0.189
1bxm	A	99	2.15	0.189
1bxr	A	1054	2.1	0.195
1bxy	A	59	1.9	0.203
1byh	A	214	2.8	0.168
1byl	A	122	2.3	0.176
1bzb	A	32	N/A	
1bzg	A	34	N/A	

1bzk	A	42	N/A	
1c0m	B	216	2.53	0.216
1c12	A	209	2.6	0.205
1c1k	A	217	1.45	0.2
1c1z	A	276	2.87	0.238
1c2a	A	120	1.9	0.191
1c2u	A	33	N/A	
1c3c	A	424	1.8	0.179
1c3g	A	125	2.7	0.265
1c3z	A	108	N/A	
1c5e	A	95	1.1	0.099
1c6w	A	33	N/A	
1c7k	A	132	1	0.148
1c7t	A	858	1.9	0.191
1c7y	A	179	3.1	0.249
1cb7	A/B	137/483	2	0.16
1cbf	A	227	2.4	0.195
1ccz	A	161	1.8	0.202
1cd3	4/B	135/54	3.5	0.275
1cd8	A	113	2.6	0.192
1ce4	A	14	N/A	
1cee	B	59	N/A	
1cew	I	104	2	0.198
1cf4	B	44	N/A	
1cf7	B	57	2.6	0.223
1cfb	A	205	2	0.202
1cfh	A	47	N/A	
1cfi	A	35	N/A	
1cfr	A	269	2.15	0.196
1cfz	A	159	2.2	0.224
1cg5	B	141	1.6	0.191
1cgi	I	53	2.3	0.195
1chd	A	198	1.75	0.182
1ci3	M	245	1.9	0.203
1ci6	A	46	2.6	0.217
1cia	A	213	2.5	0.131
1cii	A	422	3	0.241
1ciy	A	567	2.25	0.163
1cke	A	207	1.75	0.205
1ckm	A	317	2.5	0.218
1cks	C	75	2.1	0.168
1cl4	A	32	N/A	
1cm2	A	85	1.8	0.2
1cmr	A	31	N/A	
1cnv	A	283	1.65	0.172
1cnz	A	344	1.76	0.198
1co0	A	105	N/A	
1co4	A	42	N/A	
1cof	A	131	2.3	0.184
1col	A	194	2.4	0.18
1cpc	A	162	1.66	0.181
1cpm	A	209	2	0.153

1cq3	A	224	1.85	0.213
1cqx	A	401	1.75	0.182
1cqy	A	99	1.95	0.181
1cs6	A	354	1.8	0.226
1ct9	A	496	2	0.197
1ctm	A	242	2.3	0.198
1cw6	A	37	N/A	
1cwx	A	44	N/A	
1cxw	A	60	N/A	
1cyw	A	159	2.5	0.192
1cz0	A	162	2.1	0.191
1czm	A	260	2	0.186
1czq	A	45	1.5	0.214
1d06	A	130	1.4	0.224
1d0k	A	312	2.02	0.165
1d0q	A	102	1.71	0.204
1d1d	A	220	N/A	
1d2i	A	218	1.7	0.182
1d2o	A	179	2	0.212
1d2t	A	215	1.9	0.216
1d2z	C/D	104/144	2	0.211
1d3c	A	686	1.78	0.22
1d3y	B	290	2	0.197
1d4v	A	78	2.2	0.221
1d5f	A	314	2.8	0.23
1d5t	A	433	1.04	0.172
1d5y	A	216	2.7	0.254
1d66	A	57	2.7	0.23
1d6b	A	42	N/A	
1d6g	A	47	N/A	
1d7c	A	189	1.9	0.187
1d7p	M	158	1.5	0.205
1d8d	A	307	2	0.164
1d8h	A	232	2	0.229
1d9k	H	18	3.2	0.247
1dap	A	318	2.2	0.17
1daq	A	71	N/A	
1dbt	A	237	2.4	0.193
1dc1	B	310	1.7	0.19
1dce	A	510	2	0.215
1dcq	A	270	2.1	0.213
1dd3	A	128	2	0.226
1ddx	A	551	3	0.267
1ddz	A	479	2.2	0.208
1dev	B	31	2.2	0.218
1df7	A	159	1.7	0.187
1df9	A	177	2.1	0.199
1dfs	A	31	N/A	
1dfx	A	120	1.9	0.2
1dgz	A	37	N/A	
1di2	A	67	1.9	0.228
1dip	A	77	N/A	

1dj0	A	264	1.5	0.167
1dj7	A/B	109/73	1.6	0.238
1dj8	A	75	2	0.209
1dk8	A	147	1.57	0.172
1dkc	A	38	N/A	
1dlq	A	309	2.3	0.188
1dmd	A	31	N/A	
1dmg	A	166	1.7	0.208
1dml	B	35	2.7	0.23
1dmt	A	696	2.1	0.195
1dmu	A	299	2.2	0.177
1dn1	A	317	2.6	0.239
1dnw	A/D	104/465	1.9	0.197
1do6	A	124	2	0.16
1dow	B	31	1.8	0.2
1dox	A	96	N/A	
1doz	A	309	1.8	0.183
1dp3	A	55	N/A	
1dp7	P	76	1.5	0.192
1dq8	A	397	2.1	0.199
1dqc	A	73	N/A	
1dqg	A	134	1.7	0.196
1dqu	A	513	2.8	0.273
1ds1	A	323	1.08	0.135
1dsv	A	31	N/A	
1dsz	B	84	1.7	0.198
1dt9	A	41	2.7	0.246
1dto	A	198	1.9	0.232
1dtp	A	190	2.5	0.197
1dtv	A	67	N/A	
1du2	A	76	N/A	
1du5	A	206	2.5	0.167
1dus	A	192	1.8	0.188
1dvb	A	190	1.9	0.199
1dvk	B	149	2.15	0.203
1dvo	A	152	2	0.197
1dw9	A	152	1.65	0.15
1dwl	A	59	N/A	
1dx5	L	110	2.3	0.2
1dxg	A	36	1.8	0.169
1dxj	A	242	1.8	0.182
1dxy	A	329	1.86	0.196
1dy2	A	168	2	0.2
1dzb	A	203	2	0.225
1E+08	D	88	N/A	
1e0a	B	46	N/A	
1e0b	A	53	1.9	0.226
1e0c	A	270	1.8	0.18
1e0f	K	45	3.1	0.208
1e0g	A	48	N/A	
1E+12	A	239	1.8	0.237
1E+19	A	313	1.5	0.183

1e1h	D	164	1.8	0.196
1E+29	A	135	1.21	0.149
1E+39	A	568	1.8	0.181
1e3a	B	558	1.8	0.158
1e3d	B	537	1.8	0.167
1E+44	B	90	2.4	0.192
1E+58	A	246	1.25	0.121
1e5l	A	392	2.4	0.222
1e5r	B	192	2.3	0.225
1e5u	I	187	N/A	
1e5x	A	363	2.25	0.222
1e6b	A	181	1.65	0.196
1e6i	A	111	1.87	0.187
1e6y	C/E	246/433	1.6	0.16
1E+79	I	2	2.4	0.225
1e7u	A	705	2	0.254
1e8c	B	483	2	0.202
1e8i	A	108	1.95	0.248
1e8q	A	46	N/A	
1e9g	A	278	1.15	0.136
1e9k	A	38	N/A	
1e9s	A	413	2.5	0.209
1ea7	A	307	0.93	0.097
1eab	A	243	2.6	0.198
1eah	4	68	2.9	0.185
1eai	C	61	2.4	0.191
1eb0	A	139	1.85	0.22
1eb6	A	177	1	0.104
1ebt	A	142	1.9	0.184
1ecg	B	474	2.3	0.147
1eci	B	34	N/A	
1ecr	A	287	2.7	0.196
1ecz	A	140	2.68	0.195
1ed7	A	45	N/A	
1edk	A	56	N/A	
1eds	A	31	N/A	
1edv	A	34	N/A	
1edx	A	40	N/A	
1ee8	A	266	1.9	0.214
1eej	A	201	1.9	0.214
1eem	A	233	2	0.219
1eer	A/B	160/203	1.9	0.242
1ees	B	46	N/A	
1efv	A	309	2.1	0.172
1eg3	A	259	2	0.194
1egm	M	134	1.85	0.182
1ehd	A	86	1.5	0.229
1ehs	A	48	N/A	
1ei7	A	132	2.45	0.195
1eia	A	175	2.7	0.227
1eik	A	77	N/A	
1eiy	B	483	3.3	0.221

1ej6	D	417	3.6	0.206
1ej8	A	139	1.55	0.196
1ejb	A	168	1.85	0.197
1ejf	A	110	2.49	0.236
1ekj	G	214	1.93	0.229
1ekr	A	83	2	0.219
1el6	A	199	2	0.208
1elk	A	153	1.5	0.193
1elv	A	303	1.7	0.186
1em8	D	111	2.1	0.229
1emw	A	88	N/A	
1en7	A	130	2.4	0.239
1eo0	A	77	N/A	
1eo6	B	117	1.8	0.229
1eq2	F	302	2	0.212
1eq6	A	184	1.9	0.217
1eqf	A	248	2.1	0.218
1eqn	A	289	2.9	0.22
1es9	A	207	1.3	0.192
1esx	A	96	N/A	
1eto	B	93	1.9	0.225
1eu1	A	766	1.3	0.121
1eu3	A	210	1.68	0.207
1euv	A	219	1.6	0.19
1ev0	A	58	N/A	
1ewf	A	434	1.7	0.198
1ews	A	32	N/A	
1exk	A	79	N/A	
1ext	A	160	1.85	0.203
1eyf	A	92	N/A	
1eyr	A	211	2.2	0.185
1eys	C/H	300/234	2.2	0.231
1eyv	B	133	1.6	0.19
1ez1	A	388	1.75	0.189
1ezv	G	52	2.3	0.222
1ezw	A	315	1.65	0.198
1f00	I	282	1.9	0.215
1f0i	A	496	1.4	0.13
1f0x	A	497	1.9	0.209
1f13	A	657	2.1	0.183
1f1e	A	141	1.37	0.159
1f34	B	131	2.45	0.218
1f39	A	101	1.9	0.228
1f3l	A	313	2.03	0.209
1f3m	A	60	2.3	0.237
1f3u	F	128	1.7	0.225
1f5n	A	570	1.7	0.226
1f5v	A	240	1.7	0.189
1f6f	A	152	2.3	0.225
1f6v	A	91	N/A	
1f7t	A	116	1.8	0.196
1f7u	A	542	2.2	0.19

1f89	A	264	2.4	0.229
1f8p	A	36	N/A	
1f9y	A	156	0.89	0.112
1faz	A	122	1.4	0.188
1fbm	A	46	2.7	0.195
1fbv	A	303	2.9	0.227
1fc3	A	118	2	0.196
1fct	A	32	N/A	
1fcu	A	319	2.1	0.2
1fd4	A	40	1.7	0.187
1fe6	A	52	1.8	0.198
1feu	D	175	2.3	0.208
1ffq	A	540	1.9	0.189
1fft	C	163	3.5	N/A
1fgj	A	499	2.8	0.23
1fh1	A	37	N/A	
1fio	A	184	2.1	0.219
1fiu	A	286	1.6	0.173
1fj1	E	125	2.68	0.226
1fjg	Q	76	3	0.221
1fjr	A	160	2.3	0.212
1fk8	A	240	1.95	0.185
1fka	Q	84	3.3	0.304
1fkm	A	299	1.9	0.199
1flc	B	60	3.2	0.223
1flm	A	122	1.3	0.15
1fma	E	142	1.58	0.172
1fmb	A	101	1.8	0.136
1fmh	B	31	N/A	
1fn9	A	365	1.8	0.169
1fne	A	179	1.9	0.231
1fnh	A	222	2.8	0.216
1fnm	A	589	2.8	0.212
1fnt	E	132	3.2	0.255
1fo1	A	131	2.9	0.25
1foa	A	338	1.8	0.185
1fp0	A	88	N/A	
1fp1	D	334	1.82	0.226
1fpm	A	540	3	0.266
1fpo	A	170	1.8	0.216
1fqj	C	38	2.02	0.233
1fr3	A	67	1.5	0.185
1fre	A	39	N/A	
1fs0	G	212	2.1	0.216
1fs1	A	40	1.8	0.218
1fs7	A	470	1.6	0.18
1fsc	A	61	2	0.188
1fsg	A	233	1.05	0.122
1fsj	B	134	1.8	0.189
1ft5	A	207	1.6	0.188
1ftr	A	296	1.7	0.198
1fui	A	585	2.5	0.162

1fuk	A	156	1.75	0.221
1fv5	A	36	N/A	
1fvl	A	70	N/A	
1fvn	A	35	N/A	
1fw9	A	164	1.4	0.164
1fx2	A	224	1.46	0.178
1fx7	A	220	2	0.214
1fy2	A	220	1.2	0.218
1fzp	B	103	2.95	0.22
1g1t	A	157	1.5	0.196
1g1z	A	31	N/A	
1g26	A	31	N/A	
1g2b	A	59	1.12	0.148
1g2c	A/B	31/28	2.3	0.233
1g2h	A	61	N/A	
1g2r	A	91	1.35	0.157
1g31	A	83	2.3	0.225
1g38	A	393	2	0.196
1g3j	B	29	2.1	0.229
1g3m	A	290	1.7	0.193
1g3t	A	170	2.35	0.238
1g4g	A	86	N/A	
1g55	A	311	1.8	0.21
1g5g	A	304	3.3	0.224
1g60	A	234	1.74	0.198
1g61	A	225	1.3	0.132
1g65	L	208	2.25	0.283
1g66	A	205	0.9	0.107
1g6o	A	305	2.5	0.224
1g6s	A	427	1.5	0.149
1g6u	B	48	1.48	0.198
1g71	A	344	2.3	0.211
1g7o	A	215	N/A	
1g87	B	611	1.6	0.174
1g8a	A	227	1.4	0.203
1g8e	A	79	1.8	0.218
1g8k	A	822	1.64	0.154
1g8m	A	580	1.75	0.2
1g8p	A	295	2.1	0.214
1g8q	A	90	1.6	0.188
1g8t	A	240	1.1	0.129
1g96	A	91	2.5	0.22
1g9l	A	144	N/A	
1g9p	A	45	N/A	
1ga1	A	370	1.4	0.108
1gd0	A	118	1.5	0.191
1gd8	A	104	2.3	0.21
1gdt	A	179	3	0.235
1ger	B	449	1.86	0.168
1gff	2	177	3	0.352
1ggq	A	134	2.51	0.231
1ggw	A	140	N/A	

1ggx	A	216	1.9	0.192
1ggz	A	144	1.5	0.183
1gh9	A	71	N/A	
1ghc	A	75	N/A	
1ghe	B	167	1.55	0.209
1gig	H	219	2.3	0.195
1gio	A	125	N/A	
1gjj	A	93	N/A	
1gk4	A	71	2.3	0.242
1gk5	A	49	N/A	
1gk7	A	39	1.4	0.197
1gk8	A	464	1.4	0.149
1gk9	A	199	1.3	0.148
1gl0	I	6	3	0.174
1gl2	A/D	54/55	1.9	0.176
1gl4	B	89	2	0.217
1glg	A	309	2	0.167
1gme	A	127	2.7	0.231
1gmr	A	96	1.77	0.146
1gmu	C	140	1.5	0.223
1gmx	A	106	1.1	0.128
1gnf	A	46	N/A	
1go3	E/F	177/107	1.75	0.217
1go4	G	16	2.05	0.24
1goa	A	156	1.9	0.194
1got	A	326	2	0.207
1gou	A	108	1.65	0.14
1gp0	A	133	1.4	0.209
1gp8	A	40	N/A	
1gpc	A	218	2.2	0.236
1gpj	A	367	1.95	0.212
1gq5	A	91	2.2	0.222
1gqe	A	325	1.81	0.222
1gqy	B	466	1.8	0.184
1gr0	A	328	1.95	0.211
1gri	A	194	3.1	0.213
1gru	L	267	12.5	N/A
1gsa	A	314	2	0.188
1gsl	A	239	2	0.185
1gso	A	408	1.6	0.209
1gt0	D	38	2.6	0.232
1guq	A	345	1.8	0.174
1gv8	A	152	1.95	0.169
1gvj	B	139	1.53	0.208
1gvm	F	127	2.8	0.226
1gw6	A	606	2.2	0.174
1gwe	A	491	0.88	0.089
1gx3	A	274	1.7	0.209
1gxe	A	130	N/A	
1gxl	A	46	3	0.252
1gxs	D	158	2.3	0.165
1gxt	A	87	1.27	0.134

1gyo	A	106	1.2	0.13
1gyx	A	71	1.35	0.142
1gzs	B	164	2.3	0.226
1h0a	A	153	1.7	0.185
1h20	A	39	N/A	
1h21	A	239	2.5	0.199
1h2c	A	125	1.6	0.164
1h2i	A	157	2.7	0.225
1h31	A	260	2.55	0.21
1h3m	A	213	2.4	0.237
1h3o	A	43	2.3	0.226
1h4a	X	172	1.15	0.155
1h4i	D	73	1.94	0.199
1h4y	A	115	1.61	0.159
1h6f	B	186	1.7	0.201
1h6g	A	217	2.2	0.193
1h6p	A	170	2.2	0.235
1h6q	A	168	N/A	
1h7d	A	49	N/A	
1h8p	A	88	1.82	0.207
1h97	A	147	1.17	0.121
1h99	A	220	1.55	0.224
1h9e	A	56	N/A	
1h9h	I	28	1.5	0.233
1h9j	A	145	1.8	0.2
1h9t	A	121	3.25	0.264
1ha8	A	51	N/A	
1har	A	167	2.2	0.208
1hbq	A	177	1.7	0.196
1hbw	A	57	N/A	
1hcr	A	34	2.3	0.228
1hdh	A	524	1.3	0.199
1hdj	A	77	N/A	
1hfe	L	381	1.6	0.158
1hg0	A	327	1.9	0.171
1hhn	A	101	N/A	
1hhv	A	73	N/A	
1hi7	A	60	N/A	
1hkx	E	83	2.65	0.246
1hly	A	39	N/A	
1hn0	A	962	1.9	0.156
1hn3	A	40	N/A	
1hn6	A	110	N/A	
1hns	A	47	N/A	
1hnw	N	21	3.4	0.222
1hnx	I	17	3.4	0.232
1hof	A	32	N/A	
1hp8	A	68	N/A	
1hpu	A	521	1.85	0.207
1hpw	A	129	N/A	
1hq0	A	290	1.83	0.191
1hq1	A	67	1.52	0.151

1hqi	A	90	N/A	
1hqs	A	407	1.55	0.202
1hr0	J	9	3.2	0.218
1hre	A	67	N/A	
1hrt	I	65	2.8	0.155
1hru	A	181	2	0.202
1ht2	G	210	2.8	0.262
1ht9	A	76	1.76	0.162
1hth	A	31	N/A	
1htr	P	34	1.62	0.179
1htw	A	153	1.7	0.199
1hu3	A	136	2.37	0.249
1hvy	A	287	1.9	0.201
1hw5	A	191	1.82	0.228
1hxd	A	280	2.4	0.189
1hxh	A	245	1.22	0.146
1hxi	A	108	1.6	0.186
1hxm	A	174	3.12	0.219
1hxn	A	203	1.8	0.176
1hy9	A	41	N/A	
1hyp	A	73	1.8	0.187
1hyq	A	206	2.6	0.206
1hyw	A	58	N/A	
1hz4	A	356	1.45	0.189
1hz8	A	82	N/A	
1hza	A	67	1.8	0.193
1hzd	A	266	2.2	0.207
1hzf	A	276	2.3	0.215
1hzm	A	154	N/A	
1i19	A	540	1.7	0.182
1i1n	A	224	1.5	0.183
1i1q	A	483	1.9	0.219
1i1r	B	124	2.4	0.213
1i1w	A	299	0.89	0.09
1i25	A	37	N/A	
1i26	A	34	N/A	
1i27	A	73	1.02	0.126
1i2a	A	211	1.85	0.222
1i2t	A	61	1.04	0.143
1i39	A	199	1.95	0.244
1i3q	L	4	3.1	0.229
1i51	D	67	2.45	0.233
1i5p	A	628	2.2	0.183
1i6v	E	41	3.3	0.276
1i72	B	61	2	0.201
1i7d	A	499	2.05	0.233
1i7o	A	412	1.7	0.251
1i7w	D	20	2	0.206
1i7x	B	19	3	0.196
1ia9	B	242	2	0.24
1iaw	A	283	2.4	0.243
1ib9	A	34	N/A	

1iba	A	78	N/A	
1ibm	S	9	3.31	0.231
1ibw	A	75	3.2	0.204
1ibx	B	89	N/A	
1icf	B	41	2	0.182
1id0	A	133	1.6	0.199
1idu	A	566	2.24	0.177
1ie0	A	154	1.6	0.174
1ie5	A	107	N/A	
1ie6	A	33	N/A	
1ie9	A	255	1.4	0.214
1ied	A	200	2	0.226
1iez	A	217	N/A	
1ifl	A	53	5	N/A
1ig0	B	319	1.8	0.217
1ig4	A	75	N/A	
1igl	A	67	N/A	
1ihm	B	463	3.4	0.26
1ihn	A	107	2.2	0.228
1iho	A	282	1.7	0.211
1iie	A	75	N/A	
1iij	A	35	N/A	
1iio	A	84	N/A	
1ij6	A	269	3.1	0.249
1ija	A	148	N/A	
1ijf	A	440	3	0.25
1ijg	A	223	2.9	0.224
1ijy	A	122	1.35	0.225
1ijz	A	113	N/A	
1ik9	A	126	2.3	0.228
1iko	P	138	1.92	0.216
1ikp	A	586	1.45	0.205
1im2	A	269	2.8	0.233
1io0	A	166	1.45	0.203
1iod	G	32	2.3	0.201
1ioj	A	57	N/A	
1iom	A	369	1.5	0.172
1ip0	A	50	N/A	
1ipb	A	191	2	0.196
1iq4	A	173	1.8	0.215
1iqc	A	308	1.8	0.222
1iqq	A	200	1.5	0.172
1iqs	A	88	N/A	
1iqv	A	152	2.1	0.196
1iqz	A	81	0.92	0.097
1ir6	A	223	2.9	0.228
1irq	B	45	1.5	0.211
1iru	V/X	173/153	2.75	0.25
1irx	B	325	2.6	0.225
1is2	A	635	2.2	0.209
1isu	A	62	1.5	0.173
1it8	A	574	2.5	0.232

1itv	A	187	1.95	0.229
1iu4	A	329	2.4	0.199
1iua	A	83	0.8	0.092
1iuh	A	181	2.5	0.198
1iv3	A	150	1.52	0.202
1ivz	A	132	N/A	
1iw1	A	212	1.5	0.177
1iw7	C/D	607/823	2.6	0.228
1iwf	A	34	N/A	
1iwg	A	301	3.5	0.29
1iwl	A	177	1.65	0.222
1iwp	E	184	2.1	0.209
1ix5	A	151	N/A	
1ixa	A	39	N/A	
1ixk	A	298	1.9	0.199
1iyc	A	36	N/A	
1iyj	A	32	3.4	0.244
1izl	Y/Z	192/91	3.7	0.53
1izm	A	156	1.95	0.194
1izn	A	176	2.1	0.222
1j0b	S	312	2.7	0.291
1j1e	F	60	3.3	0.251
1j1v	A	92	2.1	0.229
1j25	A	133	1.78	0.228
1j26	A	112	N/A	
1j27	A	93	1.7	0.178
1j2m	A	99	N/A	
1j2r	A	188	1.3	0.145
1j30	A	141	1.7	0.192
1j31	A	255	1.6	0.166
1j34	C	34	1.55	0.183
1j3a	A	129	1.6	0.209
1j3g	A	187	N/A	
1j3m	A	127	2	0.194
1j3w	B	135	1.5	0.208
1j3x	A	77	N/A	
1j4n	A	215	2.2	0.266
1j5p	A	226	1.9	0.224
1j5s	A	406	2.85	0.234
1j5u	A	125	2	0.212
1j6r	A	168	2.3	0.251
1j6u	A	395	2.3	0.234
1j70	A	473	2.3	0.21
1j7x	A	288	1.8	0.205
1j8b	A	81	1.75	0.18
1j8f	A	312	1.7	0.235
1j8i	A	93	N/A	
1j9j	A	242	1.9	0.203
1ja1	A	599	1.8	0.208
1jad	A	207	2.4	0.254
1jat	B	128	1.6	0.203
1jax	A	205	1.8	0.185

1jb0	B/D/F/J	664/135/110/20	2.5	0.199
1jb6	A	31	1.7	0.231
1jbi	A	100	N/A	
1jc6	A	65	N/A	
1jc9	A	220	2.01	0.183
1jch	A	282	3.02	0.237
1jcz	A	260	1.55	0.194
1jd2	H	219	3	0.251
1jdb	F	376	2.1	0.179
1jdh	B	36	1.9	0.203
1jdm	A	31	N/A	
1je5	B	172	1.9	0.221
1jei	A	53	N/A	
1jej	A	351	2.5	0.197
1jek	A/B	40/34	1.5	0.213
1jeq	A	441	2.7	0.222
1jeu	A	513	1.25	0.224
1jey	B	490	2.5	0.218
1jfi	B	87	2.62	0.233
1jfl	A	228	1.9	0.194
1jfr	A	260	1.9	0.14
1jft	A	325	2.5	0.192
1jfw	A	86	N/A	
1jfx	A	217	1.65	0.152
1jg9	A	628	1.66	0.185
1jgt	B	498	1.95	0.195
1jh7	A	125	2.4	0.214
1jh8	A	346	1.8	0.175
1jhf	A	197	1.8	0.24
1jhn	A	230	2.9	0.327
1ji7	C	80	1.45	0.184
1ji8	A	111	N/A	
1ji9	A	37	N/A	
1jid	A	114	1.8	0.186
1jj2	J	129	2.4	0.189
1jjd	A	52	N/A	
1jjo	A	40	3.06	0.232
1jke	A	145	1.55	0.209
1jkv	A	266	1.39	0.104
1jl2	A	150	1.76	0.249
1jm1	A	197	1.11	0.106
1jm7	B	97	N/A	
1jma	A	247	2.65	0.236
1jmm	A	356	2.4	0.207
1jms	A	346	2.36	0.214
1jmu	A/F	23/472	2.8	0.216
1jmv	A	140	1.85	0.22
1jmz	G	76	2	0.215
1jni	A	64	1.25	0.16
1jnp	A	100	2.5	0.196
1jnr	B	148	1.6	0.18
1jo0	A	97	1.37	0.138

1jo6	A	45	N/A	
1joo	A	149	N/A	
1jos	A	98	1.7	0.216
1jou	C	38	1.8	0.222
1jp3	A	210	1.8	0.194
1jpd	X	278	2.6	0.209
1jq3	A	295	1.8	0.196
1jqn	A	744	2.35	0.195
1jr3	A	108	2.7	0.268
1jr5	A	90	N/A	
1jrf	A	47	N/A	
1jrm	A	104	N/A	
1jrr	A	357	1.6	0.197
1js8	A	382	2.3	0.202
1jsd	B	160	1.8	0.218
1jsm	A	316	1.9	0.234
1jsu	C	60	2.3	0.192
1jtg	B	165	1.73	0.173
1jv1	A	490	1.9	0.183
1jv2	A	660	3.1	0.255
1jv8	A	58	N/A	
1jvr	A	137	N/A	
1jvw	A	160	1.7	0.179
1jw2	A	72	N/A	
1jw9	B	239	1.7	0.175
1jwh	D	80	3.1	0.267
1jwq	A	179	1.8	0.176
1jx1	D	203	2.3	0.23
1jxc	A	68	N/A	
1jxy	A	46	0.89	0.145
1jy2	O	49	1.4	0.216
1jyh	A	155	1.8	0.196
1jyo	F	81	1.9	0.224
1k0h	A	117	N/A	
1k0m	B	236	1.4	0.138
1k0r	A	219	1.7	0.248
1k1f	A	63	2.2	0.262
1k20	A	310	1.5	0.175
1k24	A	178	2.03	0.235
1k28	D	42	2.9	0.211
1k2a	A	136	1	0.132
1k2f	A	153	2.6	0.22
1k2x	B	135	1.65	0.163
1k2y	X	459	1.75	0.168
1k30	A	363	1.9	0.186
1k36	A	46	N/A	
1k4i	A	216	0.98	0.178
1k4n	A	180	1.6	0.199
1k4t	A	509	2.1	0.229
1k4u	P	32	N/A	
1k51	A	72	1.8	0.186
1k5c	A	330	0.96	0.114

1k5k	A	87	N/A	
1k7v	A	170	2.2	0.232
1k7y	A	458	3	0.218
1k81	A	36	N/A	
1k87	A	440	2	0.208
1k8a	U	54	3	0.227
1k8h	A	133	N/A	
1k8k	G	102	2	0.216
1k8v	A	39	N/A	
1k8w	A	293	1.85	0.184
1k92	A	421	1.6	0.163
1k9m	3	29	3	0.219
1k9u	A	69	1.75	0.192
1ka1	A	354	1.3	0.133
1kaf	A	108	1.6	0.226
1kat	V	99	N/A	
1kb0	A	663	1.44	0.16
1kb9	D	180	2.3	0.218
1kbe	A	49	N/A	
1kbh	A/B	47/59	N/A	
1kbl	A	848	1.94	0.195
1kbt	A	60	N/A	
1kc8	O	94	3.01	0.195
1kcq	A	104	1.65	0.177
1kd1	N	138	3	0.22
1kdj	A	102	1.7	0.234
1keo	A	142	2.2	0.215
1key	A	118	2.65	0.247
1kft	A	56	N/A	
1kfu	L	699	2.5	0.221
1kg1	A	60	N/A	
1kgd	A	170	1.31	0.186
1kgs	A	208	1.5	0.179
1khc	A	137	1.8	0.19
1khx	A	201	1.8	0.215
1khy	D	136	1.95	0.222
1khz	A	203	2.04	0.196
1kil	E	8	2.3	0.245
1kjs	A	74	N/A	
1kjy	B	20	2.7	0.238
1kkd	A	92	N/A	
1kl9	A	165	1.9	0.198
1klx	A	132	1.95	0.187
1kn3	A	180	1.8	0.21
1kn7	A	75	N/A	
1knm	A	129	1.2	0.123
1knv	B	281	2.17	0.218
1kny	A	253	2.5	0.168
1knz	A	154	2.45	0.228
1ko6	A	152	3	0.247
1ko7	A	280	1.95	0.226
1koo	C	96	3.8	0.317

1kp6	A	79	1.8	0.164
1kp8	A	258	2	0.243
1kpl	D	138	3	0.254
1kpt	A	104	1.75	0.174
1kq1	H	66	1.55	0.237
1kq3	A	364	1.5	0.173
1kq6	A	127	1.18	0.132
1kqf	B	283	1.6	0.177
1kqg	C	135	2.8	0.198
1kql	A	30	2.7	0.252
1kqq	A	131	N/A	
1kqs	X	137	3.1	0.173
1kqw	A	134	1.38	0.159
1kr4	A	107	1.4	0.218
1krh	A	337	1.5	0.241
1ksf	X	684	2.6	0.216
1ksq	A	75	N/A	
1kta	A	365	1.9	0.24
1kth	A	58	0.95	0.136
1ktu	A	143	N/A	
1ktw	A	456	2	0.203
1kv4	A	42	N/A	
1kvd	A	63	1.8	0.186
1kve	B	77	1.8	0.172
1kw4	A	68	1.75	0.22
1kwa	A	88	1.93	0.251
1kwf	A	358	0.94	0.094
1kwh	A	492	2	0.185
1kwk	A	638	2.2	0.169
1kwm	A	401	1.6	0.136
1kwn	A	207	1.2	0.127
1kyo	N	381	2.97	0.229
1kyq	B	249	2.2	0.219
1l0l	F	105	2.35	0.259
1l0n	I	22	2.6	0.261
1l0o	C	51	2.9	0.221
1l0q	A	385	2.4	0.214
1l1c	A	55	N/A	
1l1d	B	143	1.85	0.207
1l1i	A	84	N/A	
1l1l	A	710	1.75	0.223
1l1o	C	75	2.8	0.236
1l1p	A	106	N/A	
1l1s	A	108	2.3	0.202
1l2f	A	244	2.5	0.223
1l2l	A	432	2	0.213
1l2p	A	61	1.55	0.282
1l2w	I	36	2	0.251
1l3a	C	140	2.3	0.216
1l3g	A	123	N/A	
1l3x	A	73	N/A	
1l3y	A	41	N/A	

1l4v	A	40	N/A	
1l5i	A	118	N/A	
1l6h	A	69	N/A	
1l6p	A	116	1.65	0.139
1l8c	B	51	N/A	
1l8d	A	101	2.2	0.225
1l8t	A	246	2.4	0.234
1l9g	A	190	2.5	0.226
1l9l	A	74	0.92	0.137
1l9m	B	665	2.1	0.182
1l9v	A	251	2.6	0.241
1l9x	B	291	1.6	0.181
1la4	A	34	N/A	
1lb6	A	155	1.8	0.203
1lbu	A	212	1.8	0.164
1lc3	A	292	1.5	0.214
1lcp	A	484	1.65	0.16
1ldj	A	224	3	0.247
1lf7	A	164	1.2	0.223
1lfp	A	216	1.72	0.228
1lfw	A	468	1.8	0.174
1lg4	A	99	N/A	
1lg7	A	160	1.96	0.208
1lgh	A	55	2.4	0.211
1lgy	A	265	2.2	0.186
1lh0	A	205	2	0.205
1lhs	A	153	2	0.182
1li1	F	225	1.9	0.182
1ljp	A	98	1.8	0.198
1lju	A	122	1.4	0.241
1ljv	A	36	N/A	
1lk9	B	427	1.53	0.193
1lkt	A	104	2.6	0.199
1lkv	X	206	2.8	0.255
1lkx	C	495	3	0.228
1llm	C	83	1.5	0.216
1llr	F	103	1.46	0.149
1lm4	A	189	1.45	0.17
1lm5	B	193	1.8	0.203
1lm8	V	142	1.85	0.196
1lmi	A	130	1.5	0.197
1lmj	A	86	N/A	
1lml	A	464	1.86	0.191
1lmp	A	129	2	0.159
1lns	A	763	2.2	0.184
1lom	A	101	1.72	0.267
1lpv	A	52	N/A	
1lqk	A	131	1.35	0.143
1lqs	M	123	2.7	0.244
1lr1	A	57	N/A	
1lr9	A	73	2.5	0.22
1lrl	A	336	1.8	0.194

1lrv	A	219	2.6	0.204
1ls3	A	434	2.7	0.22
1lsh	A	880	1.9	0.193
1lsl	A	97	1.9	0.238
1lsp	A	185	2.45	0.175
1ltl	E	180	3	0.245
1lu4	A	130	1.12	0.153
1lua	A	282	1.9	0.185
1luj	B	67	2.5	0.21
1lup	A	31	N/A	
1lv3	A	65	N/A	
1lv7	A	248	1.5	0.154
1lvo	D	275	1.96	0.21
1lw1	A	171	2.3	0.184
1lyq	B	96	1.5	0.216
1lyv	A	283	1.36	0.129
1m0v	A	130	N/A	
1m0w	A	454	1.8	0.172
1m1c	A	438	3.5	0.266
1m1f	A	107	1.4	0.16
1m1h	A	182	1.95	0.234
1m1l	A	228	2.65	0.224
1m1r	A	82	1.02	0.149
1m1s	A	107	1.8	0.278
1m21	A	487	1.8	0.203
1m2o	A	683	2.5	0.238
1m3e	B	451	2.5	0.221
1m3q	A	311	1.9	0.235
1m3y	A	413	2	0.342
1m46	A	141	2.1	0.199
1m4d	A	181	1.8	0.174
1m4j	A	133	1.6	0.232
1m56	A	544	2.3	0.236
1m5q	W	125	2	0.191
1m5y	D	278	3	0.228
1m6e	X	239	3	0.225
1m6y	A	286	1.9	0.197
1m6z	A	190	1.35	0.14
1m7v	A	340	1.95	0.225
1m8a	A	56	1.7	0.193
1m93	A	46	1.65	0.188
1m98	A	316	2.1	0.215
1m9d	D	146	1.9	0.163
1m9g	A	97	N/A	
1m9o	A	40	N/A	
1m9s	A	128	2.65	0.273
1mb4	A	369	1.84	0.192
1mb8	A	243	2.15	0.212
1mby	A	75	2	0.231
1mdr	A	357	2.1	0.151
1me3	A	215	1.2	0.103
1mec	4	62	3.2	N/A

1meo	A	202	1.72	0.223
1mex	L	213	1.25	0.153
1mg7	B	352	1.55	0.204
1mgt	A	169	1.8	0.171
1mgx	A	35	N/A	
1mhm	B	53	2.3	0.218
1mhs	A	912	8	
1mhz	D	510	2.7	0.152
1mid	A	90	1.71	0.163
1miu	A	38	3.1	0.256
1mix	A	206	1.75	0.199
1mj4	A	80	1.2	0.118
1mjh	B	137	1.7	0.21
1mkc	A	43	N/A	
1mkn	A	59	N/A	
1mky	A	400	1.9	0.204
1mkz	B	169	1.6	0.183
1ml4	A	307	1.8	0.172
1ml8	A	130	2.6	0.228
1ml9	A	246	1.98	0.205
1mla	A	305	1.5	0.184
1mlw	A	290	1.71	0.205
1mm2	A	61	N/A	
1mm9	A	126	1.66	0.133
1mms	A	71	2.57	0.219
1mn8	D	97	1	0.133
1mnm	A	78	2.25	0.24
1mnn	A	290	1.4	0.195
1mo1	A	84	1.8	0.164
1mo8	A	213	N/A	
1mof	A	53	1.7	0.169
1mog	A	67	1.7	0.18
1mpe	A	56	N/A	
1mph	A	106	N/A	
1mpz	A	41	N/A	
1mqq	A	674	1.65	0.185
1mr4	A	47	N/A	
1msw	D	365	2.1	0.241
1mtp	B	35	1.5	0.195
1mu2	A	453	2.35	0.192
1mus	A	449	1.9	0.191
1mv4	A	37	N/A	
1mw5	A	151	2.1	0.196
1mwk	A	304	2.3	0.217
1mwp	A	96	1.8	0.203
1mwq	A	99	0.99	0.108
1mx0	C	461	2.3	0.214
1mx9	L	450	2.9	0.214
1mz8	A	87	2	0.183
1mzb	A	132	1.8	0.226
1mzg	A	140	2	0.208
1mzw	B	31	2	0.195

1n07	B	140	2.45	0.228
1n0e	F	148	2.7	0.231
1n0r	A	126	1.5	0.216
1n0w	A/B	201/32	1.7	0.191
1n1c	A	179	2.4	0.224
1n26	A	247	2.4	0.22
1n2d	C	45	2	0.208
1n2m	C	162	1.9	0.186
1n3h	A	207	N/A	
1n3j	A	119	N/A	
1n3l	A	317	1.18	0.18
1n40	A	395	1.06	0.133
1n48	A	291	2.2	0.204
1n4k	A	281	2.2	0.224
1n4n	A	47	N/A	
1n4w	A	467	0.92	0.103
1n4x	L	113	1.7	0.227
1n54	A	338	2.72	0.263
1n5g	A	38	N/A	
1n5i	A	208	1.85	0.21
1n5u	A	574	1.9	0.235
1n61	C	287	1.3	0.142
1n62	A	161	1.09	0.144
1n63	B	805	1.21	0.149
1n6f	A	998	2.7	0.247
1n6j	A	82	2.2	0.243
1n6z	A	105	N/A	
1n7e	A	95	1.5	0.254
1n7l	A	53	N/A	
1n7u	A	498	2.4	0.224
1n80	A	324	2.45	0.236
1n81	A	167	2.1	0.207
1n8l	A	76	N/A	
1n8m	A	38	N/A	
1n8p	A	294	2.6	0.253
1n8w	A	655	2.7	0.19
1n8y	C	592	2.4	0.224
1n8z	C	581	2.52	0.222
1n93	X	330	1.76	0.163
1n9e	A	731	1.65	0.161
1n9p	A	189	1.8	0.231
1na6	B	380	2.1	0.23
1nay	A	54	2.6	0.234
1nba	A	252	2	0.186
1nbc	A	155	1.75	0.193
1nbu	A	118	1.6	0.177
1nbw	A	573	2.4	0.232
1nc7	D	110	1.55	0.17
1ncr	A	279	2.7	0.243
1ncs	A	47	N/A	
1ncy	A	162	2.1	0.13
1nd7	A	318	2.1	0.243

1nd9	A	49	N/A	
1ne5	A	42	N/A	
1nee	A	135	N/A	
1nei	A	60	N/A	
1neq	A	74	N/A	
1nex	B	427	2.7	0.239
1nf8	A	207	1.6	0.134
1ng6	A	148	1.4	0.21
1ng7	A	60	N/A	
1ng9	A	794	2.6	0.215
1ngk	H	126	2.11	0.186
1ngm	B	72	2.95	0.276
1nh1	A	225	2.2	0.222
1nh2	A/D	177/103	1.9	0.201
1nh8	A	276	1.8	0.198
1nhk	R	144	1.9	0.174
1nhm	A	79	N/A	
1nho	A	85	N/A	
1ni0	C	127	2.5	0.228
1ni4	B	317	1.95	0.202
1ni5	A	299	2.65	0.223
1nig	A	142	2	0.211
1nij	A	302	2	0.223
1nj1	A	159	2.55	0.225
1njh	A	101	1.7	0.22
1njq	A	37	N/A	
1nkg	A	508	1.5	0.164
1nks	A	136	2.57	0.164
1nkz	A	52	2	0.169
1nl0	G	32	2.2	0.233
1nl3	A	757	2.8	0.193
1nlf	A	254	1.95	0.201
1nlq	A	105	1.5	0.234
1nmp	A	247	2.2	0.212
1nmv	A	163	N/A	
1nn7	A	105	2.1	0.23
1nnh	A	293	1.65	0.171
1nni	1	143	2.5	0.221
1nnu	D	59	2.5	0.176
1nnv	A	107	N/A	
1no4	A	62	2.2	0.239
1no5	B	102	1.8	0.2
1no7	A	400	2.9	0.255
1nof	A	383	1.42	0.102
1nog	A	139	1.55	0.197
1nol	A	607	2.4	0.181
1nox	A	196	1.59	0.182
1npe	B	164	2.3	0.223
1nr3	A	122	N/A	
1nrb	A	63	N/A	
1nri	A	235	1.9	0.212
1nrj	A	138	1.7	0.194

1nrv	A	100	1.65	0.224
1nsj	A	196	2	0.192
1nt2	B	197	2.9	0.257
1ntv	A	152	1.5	0.222
1nty	A	288	1.7	0.195
1nu7	D	275	2.2	0.209
1nui	A	242	2.9	0.235
1nvi	D	81	1.9	0.175
1nvm	B	312	1.7	0.189
1nvp	B	26	2.1	0.229
1nw1	A	364	2.02	0.209
1nwa	A	168	1.5	0.16
1nxh	A	104	2.8	0.226
1ny1	A	231	1.8	0.182
1ny9	A	94	N/A	
1nyc	A	110	1.4	0.201
1nyh	A	64	3.1	0.271
1nyt	A	271	1.5	0.132
1nz0	D	109	1.2	0.163
1nzi	B	155	1.5	0.216
1nzj	A	273	1.5	0.146
1nzo	A	352	1.85	0.208
1o0c	A	492	2.7	0.213
1o0w	B	234	2	0.195
1o1d	E	145	70	N/A
1o1w	A	138	N/A	
1o1x	A	142	1.9	0.117
1o22	A	146	2	0.182
1o26	B	220	1.6	0.203
1o3u	A	119	1.75	0.179
1o54	A	265	1.65	0.158
1o58	A	293	1.8	0.157
1o6a	A	85	1.85	0.171
1o6d	A	147	1.66	0.229
1o6g	A	710	1.4	0.2
1o6s	B	98	1.8	0.168
1o6v	A	325	1.5	0.145
1o6w	A	75	N/A	
1o70	A	271	2.6	0.225
1o71	A	174	2.26	0.215
1o75	B	332	1.95	0.205
1o7d	B/C/E	73/149/108	2.7	0.257
1o7f	A	255	2.5	0.244
1o7i	A	115	1.2	0.191
1o7m	A	448	1.75	0.178
1o7q	A	287	1.3	0.107
1o83	A	70	1.64	0.193
1o8a	A	575	2	0.18
1o8b	B	206	1.25	0.224
1o8r	A	94	N/A	
1o8t	A	23	N/A	
1o97	D	315	1.6	0.201

1o98	A	470	1.4	0.19
1o9a	A	93	N/A	
1o9t	A	364	2.9	0.265
1oa8	D	133	1.7	0.21
1oaa	A	258	1.25	0.2
1oac	B	705	2	0.162
1oae	A	124	1.95	0.185
1oaf	A	250	1.4	0.161
1oao	C	729	1.9	0.147
1oaw	A	48	N/A	
1ob8	B	127	1.8	0.197
1obf	P	334	1.7	0.166
1obg	A	298	2.05	0.166
1occ	T	64	2.8	0.201
1ocy	A	198	1.5	0.143
1ocz	Y	43	2.9	0.195
1od2	A	681	2.7	0.226
1od6	A	153	1.5	0.196
1odh	A	114	2.85	0.219
1odz	A	379	1.4	0.151
1ofc	X	267	1.9	0.218
1ofn	A	202	1.5	0.144
1oga	A	270	1.4	0.218
1oge	A	113	2.05	0.194
1ohm	A	44	N/A	
1ohq	A	120	2	0.182
1ohu	B	149	2.03	0.212
1ohz	B	56	2.2	0.21
1oi0	A	108	1.5	0.213
1oi2	A	333	1.75	0.172
1oi7	A	270	1.23	0.178
1oio	A	178	1.7	0.216
1ois	A	221	1.9	0.222
1oiz	A	266	1.88	0.195
1ojh	D	50	1.8	0.182
1ojr	A	273	1.35	0.111
1ok0	A	65	0.93	0.103
1okb	A	222	1.9	0.186
1okc	A	269	2.2	0.22
1oke	A	105	2.4	0.263
1okg	A	305	2.1	0.208
1oks	A	51	1.8	0.198
1olm	E	397	1.95	0.18
1olt	A	371	2.07	0.154
1olz	A	562	2	0.206
1oma	A	48	N/A	
1oms	C	106	2.3	0.208
1omw	G	60	2.5	0.202
1on2	A	135	1.61	0.22
1on3	E	519	1.9	0.168
1ong	A	263	1.1	0.141
1onl	A	127	2.5	0.186

1oo4	A	111	N/A	
1ooh	A	126	1.25	0.16
1op4	A	136	N/A	
1ops	A	64	2	0.204
1oqj	A	90	1.55	0.194
1oqv	A	170	1.3	0.113
1oqw	A	144	2	0.223
1or7	A/C	181/66	2	0.197
1ord	A	730	3	0.219
1orq	C	11	3.2	0.253
1ors	C	94	1.9	0.231
1oru	A	170	1.8	0.196
1orw	A	692	2.84	0.187
1ory	B	29	2.45	0.215
1os1	A	519	1.8	0.2
1os6	A	71	1.45	0.182
1osf	A	215	1.75	0.184
1osl	A	62	N/A	
1osy	A	114	1.7	0.182
1otf	A	59	1.9	0.202
1ou0	B	165	2.1	0.202
1oul	A	103	2.2	0.256
1oup	A	213	2.3	0.191
1ouw	C	151	1.37	0.153
1ova	A	384	1.95	0.169
1ovn	A	223	1.9	0.216
1ovy	A	97	N/A	
1ow1	A	167	1.8	0.214
1ow4	A	120	1.6	0.174
1ox0	A	414	1.3	0.138
1oxj	A	169	1.8	0.229
1oxw	C	350	2.2	0.22
1oy3	C	127	2.05	0.218
1oyw	A	497	1.8	0.193
1oz2	A	324	1.55	0.189
1oz9	A	141	1.89	0.205
1ozj	A	102	2.4	0.21
1p0a	A	44	N/A	
1p0s	L	73	2.8	0.207
1p0y	B	209	2.55	0.228
1p0z	A	124	1.6	0.168
1p1j	A	523	1.7	0.165
1p27	A	144	2	0.22
1p30	A	871	2.5	0.158
1p32	A	163	2.25	0.173
1p3c	A	212	1.5	0.153
1p3q	Q	42	1.7	0.26
1p4e	B	301	2.7	0.236
1p4o	B	314	1.5	0.209
1p4p	A	135	2	0.211
1p4q	A	52	N/A	
1p4x	A	227	2.2	0.24

1p57	A	107	1.75	0.186
1p5u	B	146	1.99	0.166
1p68	A	102	N/A	
1p6o	B	161	1.14	0.11
1p6t	A	139	N/A	
1p6x	A	326	2	0.187
1p71	A	94	1.9	0.244
1p7a	A	37	N/A	
1p8a	A	146	N/A	
1p8b	A	37	N/A	
1p8c	E	104	2.3	0.225
1p8l	A	273	2.95	0.263
1p90	A	121	1.8	0.188
1p94	A	76	N/A	
1p9j	A	54	N/A	
1p9k	A	79	N/A	
1p9p	A	173	2.5	0.196
1paq	A	155	2.3	0.243
1pb5	A	35	N/A	
1pb6	A	190	2.5	0.238
1pb8	A	282	1.45	0.204
1pba	A	66	N/A	
1pbj	A	111	1.4	0.192
1pbt	A	215	1.7	0.179
1pbw	B	190	2	0.184
1pby	A/C	485/77	1.7	0.191
1pc4	A	106	1.65	0.159
1pc6	A	70	2.51	0.234
1pcf	A	66	1.74	0.197
1pcn	A	93	N/A	
1pdo	A	129	1.7	0.189
1pdy	A	409	2.4	0.215
1pe4	A	67	N/A	
1pee	A	180	1.5	0.186
1peh	A	33	N/A	
1pfb	A	55	1.4	0.197
1pfk	A	298	2.4	0.165
1pfl	A	139	N/A	
1pft	A	50	N/A	
1pfv	A	547	1.7	0.186
1pg4	B	639	1.75	0.184
1pg6	A	202	1.7	0.213
1pgx	A	68	1.66	0.191
1ph5	B	155	2.3	0.228
1pj6	A	828	1.65	0.156
1pjq	B	429	2.21	0.235
1pjx	A	308	0.85	0.121
1pjz	A	201	N/A	
1pk0	C	316	3.3	0.264
1pkl	G	426	2.35	0.209
1pkp	A	143	2.8	0.22
1plk	A	137	2.8	0.228

1pm3	A	58	3.15	0.255
1pm6	A	72	N/A	
1pmc	A	36	N/A	
1pmi	A	440	1.7	0.184
1pmj	X	185	1.55	0.124
1pnf	A	314	2	0.197
1po6	A	183	2.1	0.234
1poc	A	133	2	0.192
1poz	A	130	N/A	
1pp8	P	24	3.05	0.269
1ppo	A	216	1.8	0.155
1ppr	N	305	2	0.179
1pq1	B	33	1.65	0.219
1pq8	A	203	1	0.128
1pqf	A	122	2	0.165
1pqx	A	91	N/A	
1prb	A	53	N/A	
1pre	B	451	2.8	0.208
1prv	A	56	N/A	
1prz	A	242	1.8	0.218
1ps3	A	1014	1.8	0.2
1psm	A	38	N/A	
1psp	A	105	2.5	0.2
1psr	A	97	1.05	0.107
1ptr	A	50	2.2	0.194
1pu5	A	163	1.9	0.214
1pu6	B	216	1.64	0.151
1puj	A	255	2	0.216
1puo	B	142	1.85	0.201
1puz	A	82	N/A	
1pv1	A	290	2.3	0.255
1pv5	A	245	1.75	0.198
1pvj	A	339	3	0.227
1pw4	A	260	3.3	0.296
1px5	B	339	1.74	0.187
1pxe	A	48	N/A	
1pxf	A	111	1.87	0.149
1pyi	A	88	3.2	0.245
1pyt	A	94	2.35	0.192
1pyv	A	53	N/A	
1pz4	A	111	1.35	0.187
1pzw	A	56	2	0.241
1q05	B	127	2.2	0.208
1q08	A	94	1.9	0.183
1q0v	A	81	N/A	
1q16	C	223	1.9	0.202
1q23	B	215	2.18	0.192
1q27	A	171	N/A	
1q38	A	89	N/A	
1q3j	A	36	N/A	
1q3v	A	225	2.91	0.247
1q4k	C	162	2.3	0.245

1q50	A	540	2.6	0.195
1q51	B	252	2.3	0.202
1q5r	J	215	3.1	0.229
1q5w	A	31	N/A	
1q5z	A	145	1.8	0.194
1q60	A	99	N/A	
1q68	A	38	N/A	
1q6z	A	524	1	0.122
1q79	A	448	2.15	0.205
1q7h	A	142	2.1	0.169
1q7l	B	88	1.4	0.133
1q7t	A	303	1.9	0.19
1q81	2/D	56/185	2.95	0.21
1q82	P	99	2.98	0.207
1q86	M	65	3	0.234
1q88	B	148	2.42	0.19
1q8c	A	132	2	0.254
1q8d	A	99	1.8	0.193
1q8f	A	308	1.7	0.153
1q8h	A	34	2	0.255
1q8i	A	704	2	0.197
1q8k	A	300	N/A	
1q8r	A	118	1.9	0.19
1q90	R	31	3.1	0.222
1q92	A	195	1.4	0.133
1q95	L	12	2.46	0.23
1q9b	A	42	1.5	0.128
1q9s	A	144	2.42	0.199
1qa4	A	56	N/A	
1qab	E	141	3.2	0.278
1qbf	A	36	N/A	
1qcn	B	404	1.9	0.191
1qcs	A	190	1.9	0.21
1qcz	A	159	1.5	0.186
1qdd	A	144	1.3	0.132
1qdp	A	42	N/A	
1qdv	A	96	1.6	0.238
1qf6	A	497	2.9	0.195
1qfd	A	32	N/A	
1qfh	A	196	2.2	0.222
1qfq	B	35	N/A	
1qge	E	97	1.7	0.187
1qgi	A	259	1.6	0.192
1qgk	B	29	2.5	0.221
1qgu	D	519	1.6	0.156
1qh4	A	369	1.41	0.134
1qh5	A	260	1.45	0.204
1qhd	A	397	1.95	0.188
1qhh	C/D	84/93	2.5	0.228
1qhk	A	47	N/A	
1qhl	A	197	2.2	0.217
1qhp	A	686	1.7	0.153

1qht	A	674	2.1	0.245
1qjk	A	36	N/A	
1qjz	B	188	3.8	0.326
1qk7	A	32	N/A	
1qkd	A	61	1.49	0.169
1qkl	A	127	N/A	
1qkr	A	172	1.8	0.2
1qks	A	559	1.28	0.185
1qlb	B	238	2.33	0.213
1qld	A	50	N/A	
1qlm	A	316	2	0.198
1qlo	A	34	N/A	
1qmd	A	362	2.2	0.198
1qmg	A	514	1.6	0.196
1qmr	A	159	2.15	0.196
1qmv	A	195	1.7	0.192
1qox	N	449	2.7	0.255
1qoy	A	303	2	0.198
1qp6	A	35	N/A	
1qpq	A	283	2.45	0.178
1qqg	B	209	2.3	0.191
1qqp	4-Feb	208/45	1.9	0.161
1qqr	A	119	2.3	0.197
1qqw	B	500	2.75	0.206
1qqy	A	129	1.85	0.178
1qs1	A	402	1.5	0.235
1qsa	A	618	1.65	0.168
1qtn	B	90	1.2	0.166
1qu2	A	733	2.2	0.239
1qu9	A	126	1.2	0.164
1qun	B	273	2.8	0.24
1qvg	2	35	2.9	0.201
1qvi	A	696	2.54	0.212
1qvr	A	139	3	0.263
1qw2	A	97	1.5	0.203
1qw9	A	497	1.2	0.16
1qwd	A	167	1.75	0.169
1qwy	A	234	1.3	0.163
1qx0	A	277	2.26	0.234
1qx2	A	75	1.44	0.156
1qxf	A	58	N/A	
1qxy	A	249	1.04	0.142
1qy7	A	112	2	0.199
1qyn	D	112	2.35	0.234
1qz2	A	203	3	0.23
1qze	A	214	N/A	
1qzm	A	94	1.9	0.179
1qzn	A	171	1.9	0.159
1qzr	A	378	1.9	0.196
1r02	A	33	N/A	
1r0d	A	178	1.9	0.224
1r0s	A	251	2	0.229

1r0u	A	142	1.75	0.185
1r1f	A	37	N/A	
1r1m	A	132	1.9	0.213
1r26	A	113	1.4	0.168
1r2a	A	46	N/A	
1r2m	A	68	1	0.137
1r2q	A	165	1.05	0.124
1r3b	A	202	N/A	
1r3d	A	253	1.9	0.179
1r3i	C	99	2.4	0.217
1r44	A	202	2.25	0.254
1r48	A	33	N/A	
1r4p	D	70	1.77	0.151
1r4x	A	273	1.9	0.173
1r5e	A	105	N/A	
1r5i	H	194	2.6	0.238
1r5s	A	132	N/A	
1r5t	A	135	2	0.188
1r5z	A	320	1.95	0.191
1r6f	A	272	2.17	0.217
1r6m	A	231	2	0.226
1r6o	C	87	2.25	0.19
1r71	B	81	2.2	0.195
1r75	A	109	1.86	0.201
1r79	A	84	N/A	
1r7a	A	503	1.77	0.164
1r7h	A	74	2.69	0.243
1r7l	B	107	2	0.223
1r8g	B	348	2.15	0.189
1r8h	A	78	1.9	0.191
1r8m	E	194	1.7	0.192
1r8o	A/B	96/71	1.83	0.168
1r8u	B	100	N/A	
1r95	A	87	2.65	0.232
1r9f	A	113	1.85	0.212
1r9q	A	309	2.05	0.172
1r9w	A	135	1.8	0.213
1r9z	A	423	1.32	0.166
1ra4	A	116	1.86	0.173
1rcq	A	356	1.45	0.149
1rdu	A	116	N/A	
1re0	B	189	2.4	0.209
1re6	A	94	N/A	
1reg	X	122	1.9	0.18
1rep	C	210	2.6	0.213
1rer	A	242	3.2	0.265
1rf8	B	100	N/A	
1rgr	A	93	N/A	
1rgv	A	66	2.9	0.197
1rgx	C	94	1.79	0.204
1rh1	A	490	2.5	0.197
1rhc	A	329	1.8	0.158

1rhf	A	169	1.96	0.209
1rhh	B	221	1.9	0.224
1rhy	A	158	2.3	0.189
1rhz	A	50	3.5	0.254
1ri0	A	100	N/A	
1ri9	A	77	N/A	
1rif	A	282	2	0.218
1rin	D	48	2.6	0.183
1rip	A	81	N/A	
1riy	A	71	1.8	0.216
1rj1	A	148	1.87	0.194
1rj9	A	277	1.9	0.206
1rji	A	31	N/A	
1rjj	A	111	N/A	
1rk4	A	213	1.79	0.176
1rk5	A	474	1.8	0.183
1rk8	C	32	1.9	0.235
1rki	A	101	1.6	0.184
1rkl	A	36	N/A	
1rkq	A	271	1.4	0.177
1rkt	A	195	1.95	0.208
1rku	A	206	1.47	0.189
1rl0	A	255	1.4	0.184
1rl2	B	132	2.3	0.203
1rl6	A	162	2	0.257
1rlk	A	116	1.95	0.212
1rlv	A	296	3	0.229
1rmd	A	115	2.1	0.209
1rmk	A	31	N/A	
1rni	A	158	1.85	0.243
1ro3	A	49	N/A	
1ro7	A	239	1.8	0.217
1roc	A	155	1.5	0.198
1rod	A	72	N/A	
1rp3	A/B	199/36	2.3	0.242
1rp4	A	253	2.2	0.217
1rp5	A	403	3	0.231
1rp7	A	800	2.09	0.192
1rpy	B	85	2.3	0.228
1rq9	A	99	2.6	0.254
1rqg	A	460	2.9	0.244
1rr7	A	53	2.2	0.252
1rre	E	187	1.75	0.205
1rrz	A	66	N/A	
1rsy	A	128	1.9	0.186
1rtg	A	198	2.6	0.179
1rtq	A	291	0.95	0.135
1rtt	A	174	1.28	0.211
1rtu	A	113	1.8	0.141
1ru4	A	400	1.6	0.167
1ru8	A	199	2.7	0.218
1ruw	A	69	1.8	0.173

1rv9	A	242	1.53	0.197
1rvb	A	237	2.1	0.165
1rvk	A	370	1.7	0.193
1rw1	A	111	1.02	0.129
1rw2	A	152	N/A	
1rws	A	68	N/A	
1rwt	F	218	2.72	0.194
1rwx	A	169	1.85	0.214
1rxq	B	172	1.7	0.186
1rxx	C	381	2.45	0.198
1ry3	A	64	N/A	
1ry6	A	304	1.6	0.205
1ry9	A	133	1.82	0.181
1rya	A	160	1.3	0.169
1ryk	A	69	N/A	
1ryl	A	157	1.6	0.212
1ryp	1/G/R	219/221/199	1.9	0.286
1ryu	A	79	N/A	
1rz2	A	214	1.6	0.225
1rz4	A	207	2.1	0.187
1rzj	G	273	2.2	0.262
1rzs	A	61	N/A	
1rzw	A	123	N/A	
1s0g	A	1288	2.6	0.21
1s12	A	89	2	0.214
1s1h	B/I/J/K/L/Q/S	40/29/10/36/20/17/14	11.7	N/A
1s1i	D/E/F/I/L/M/O/P/T/U/V/W/Y/Z	9/4/8/3/2/5/5/6/2/2/5/4/2/2	11.7	N/A
1s20	G	320	2.2	0.191
1s28	A	107	3	0.248
1s29	A	89	1.6	0.232
1s2d	B	164	2.1	0.229
1s2e	A	269	2.3	0.239
1s2h	A	205	N/A	
1s2o	A	244	1.4	0.182
1s2x	A	171	1.9	0.229
1s35	A	208	2.4	0.222
1s3a	A	85	N/A	
1s3h	A	471	2.5	0.205
1s3i	A	307	2.3	0.242
1s3p	A	109	2	0.181
1s3r	A	470	2.6	0.229
1s4m	A	245	2.1	0.212
1s4n	A	337	2.01	0.18
1s4x	A	47	N/A	
1s4y	B/C	111/95	2.3	0.206
1s4z	A	75	N/A	
1s5e	A	232	1.9	0.167
1s5l	B/C/E/H/T/U/X	208/130/7/11/21/35/1	3.5	0.296
1s62	A	102	N/A	
1s6d	A	103	N/A	
1s6i	A	182	N/A	
1s72	H/M/X/Z	123/194/72/69	2.4	0.188

1s7e	A	147	N/A	
1s7i	A	124	1.8	0.218
1s7z	A	101	1.83	0.17
1s8e	A	273	2.3	0.231
1s8n	A	190	1.48	0.203
1s9h	A/C	245/102	2.4	0.227
1s9u	A	198	1.38	0.161
1sa3	A	262	1.95	0.223
1sal	A	42	N/A	
1sau	A	105	1.12	0.109
1sb0	A	87	N/A	
1sb7	B	307	2.2	0.216
1sbx	A	96	1.65	0.17
1sbz	D	174	2	0.176
1scy	A	31	N/A	
1sdi	A	213	1.65	0.15
1sdo	A	192	1.85	0.22
1sdw	A	282	1.85	0.197
1se0	A	96	1.75	0.212
1se8	A	205	1.8	0.217
1sed	A	106	2.1	0.189
1seg	A	64	1.3	0.153
1sei	A	130	1.9	0.222
1sen	A	133	1.2	0.162
1ses	A	399	2.5	0.176
1sez	A	231	2.9	0.227
1sf8	G	104	2.6	0.223
1sfd	A	105	0.99	0.116
1sg4	B	256	1.3	0.157
1sgg	A	67	N/A	
1sgm	A	184	2	0.233
1sh1	A	48	N/A	
1sh8	A	153	1.5	0.197
1shs	A	84	2.9	0.216
1shu	X	181	1.5	0.187
1si2	A	90	2.6	0.221
1sig	A	300	2.6	0.218
1siq	A	382	2.1	0.198
1sis	A	35	N/A	
1six	A	147	1.3	0.12
1sj1	A	60	1.5	0.194
1sj8	A	297	2.6	0.258
1sjw	A	142	1.35	0.143
1sk8	A	435	1.65	0.175
1skn	P	64	2.5	0.22
1skq	A	414	1.8	0.219
1skz	A	104	1.9	0.217
1slq	A	77	3.2	0.308
1sm7	A	109	N/A	
1smb	A	131	1.55	0.188
1snl	A	99	N/A	
1sox	A	449	1.9	0.175

1soy	A	106	N/A	
1sp4	A	47	2.2	N/A
1sp8	D	386	2	0.275
1sqe	B	106	1.5	0.203
1sqj	B	767	2.2	0.217
1sr6	B	69	2.75	0.242
1sr8	A	279	1.9	0.209
1sr9	B	562	2	0.169
1srq	A/D	330/66	2.9	0.232
1srv	A	144	1.7	0.199
1ss1	A	62	N/A	
1ss3	A	50	N/A	
1ssf	A	123	N/A	
1ssl	A	48	N/A	
1ssq	D	257	1.85	0.198
1ssz	A	34	N/A	
1st4	A	285	2.02	0.201
1stf	I	98	2.37	0.19
1stz	A	323	2.2	0.215
1su3	B	405	2.2	0.223
1su8	A	633	1.1	0.146
1sum	B	224	2	0.217
1suu	A	293	1.75	0.194
1suw	A	167	2.45	0.211
1sv0	C	79	2.07	0.232
1sva	2	284	3.1	0.257
1svf	A	64	1.4	0.181
1svl	A	295	1.95	0.236
1sx6	A	185	1.95	0.192
1sxr	A	173	1.56	0.177
1sy6	H	219	2.1	0.211
1syr	A	103	2.95	0.197
1szh	A	146	1.5	0.209
1szi	A	177	2.8	0.236
1szn	A	417	1.54	0.151
1szq	A	406	2.7	0.204
1t06	A	235	1.9	0.196
1t07	A	72	1.8	0.192
1t0f	A/C	261/47	1.85	0.211
1t0g	A	109	N/A	
1t0h	B	180	1.97	0.185
1t0t	Z	243	1.75	0.156
1t0z	A	72	2.6	0.2
1t11	A	212	2.5	0.239
1t15	A	211	1.85	0.206
1t16	A	190	2.6	0.257
1t1e	A	473	1.18	0.213
1t1j	B	125	1.7	0.202
1t2e	A	311	1.85	0.156
1t2k	A	50	3	0.257
1t2t	A	96	2.5	0.238
1t3g	A	142	2.3	0.228

1t3k	A	132	N/A	
1t3t	A	1278	1.9	0.188
1t3v	A	124	N/A	
1t3w	B	65	2.8	0.269
1t43	A	239	3.2	0.289
1t4e	A	94	2.6	0.239
1t4h	B	193	1.8	0.188
1t4l	B	90	N/A	
1t50	A	58	N/A	
1t5l	A	335	2.6	0.23
1t62	B	158	3	0.215
1t6a	A	119	2.05	0.194
1t6b	X	676	2.5	0.207
1t6c	A	306	1.53	0.162
1t6g	A	368	1.8	0.158
1t6t	2	108	1.8	0.18
1t6u	A	117	1.3	0.158
1t77	A	381	2.4	0.21
1t84	A	107	N/A	
1t8e	A	639	2.54	0.22
1t8k	A	76	1.1	0.133
1t8u	B	260	1.95	0.199
1t92	A	108	1.6	0.167
1t94	A	347	2.4	0.246
1t95	A	208	1.9	0.212
1t9g	C	382	2.9	0.191
1t9h	A	281	1.6	0.144
1t9i	B	155	1.6	0.194
1tab	I	36	2.3	0.2
1tac	A	86	N/A	
1taz	A	311	1.77	0.182
1tba	A	67	N/A	
1tbo	A	66	N/A	
1tc5	A	186	1.93	0.199
1td6	A	263	2.5	0.244
1te2	A	208	1.76	0.174
1te6	A	434	1.8	0.206
1tej	B	59	1.9	0.213
1ten	A	90	1.8	0.196
1tex	D	238	2.6	0.218
1tf6	D	75	3.1	0.308
1tff	A	211	2.1	0.188
1tfg	A	107	1.95	0.194
1tfi	A	35	N/A	
1tfq	A	117	N/A	
1tfr	A	282	2.06	0.205
1tgj	A	112	2	0.175
1tgs	I	52	1.8	0.186
1th1	C	13	2.5	0.225
1thx	A	108	1.6	0.175
1tig	A	88	2	0.207
1tii	C	36	2.25	0.191

1tiu	A	89	N/A	
1tiv	A	86	N/A	
1tjf	B	178	2.21	0.197
1tjg	H	233	2	0.198
1tjl	A	113	2	0.228
1tjx	A	157	1.04	0.166
1tjy	A	316	1.3	0.156
1tk4	A	118	1.1	0.168
1tlj	A	160	2.8	0.223
1tlo	A	317	1.9	0.238
1tm9	A	137	N/A	
1tmf	4	31	3.5	N/A
1tmi	A	110	1.7	0.146
1tml	A	286	1.8	0.184
1tmz	A	32	N/A	
1tnt	A	76	N/A	
1to4	A	156	1.55	0.157
1to6	A	288	2.5	0.226
1tol	A	155	1.85	0.228
1tov	A	98	1.77	0.211
1tp4	A	59	N/A	
1tp6	A	126	1.5	0.185
1tpg	A	91	N/A	
1tq1	A	119	N/A	
1tq6	A	310	2.7	0.204
1tqg	A	71	0.98	0.179
1tqh	A	242	1.63	0.168
1tqq	A	326	2.75	0.264
1tr9	A	318	1.8	0.17
1ts9	A	93	1.7	0.204
1tsp	A	544	2	0.184
1ttb	A	115	1.7	0.157
1ttf	A	94	N/A	
1ttu	A	275	2.85	0.201
1tu1	A	144	1.95	0.205
1tu2	A	105	N/A	
1tua	A	189	1.5	0.208
1tuc	A	60	2.02	0.214
1tue	D	202	2.1	0.219
1tul	A	101	2.2	0.181
1tuz	A	118	N/A	
1tv8	A	327	2.2	0.213
1tvt	A	75	N/A	
1tvv	A	283	2.3	0.209
1tw6	B	95	1.71	0.155
1twc	E	111	3	0.234
1twf	F/J	68/56	2.3	0.247
1twj	A	77	2.5	0.248
1txb	A	73	N/A	
1txg	A	335	1.7	0.149
1txj	A	152	2	0.199
1txl	A	188	1.7	0.228

1txo	B	231	1.95	0.199
1txu	A	182	2.35	0.227
1tyk	A	34	N/A	
1tzb	A	300	1.16	0.149
1tzw	A	137	1.6	0.187
1u00	A	219	1.95	0.174
1u04	A	507	2.25	0.227
1u07	A	90	1.13	0.139
1u09	A	476	1.91	0.21
1u0k	A	281	1.5	0.167
1u0s	A	84	1.9	0.227
1u10	C	235	2.4	0.234
1u14	A	167	1.68	0.182
1u19	A	270	2.2	0.2
1u26	A	314	2.5	0.207
1u2c	A	220	2.3	0.208
1u2l	A	307	2.3	0.175
1u2m	C	98	2.3	0.232
1u2z	C	349	2.2	0.213
1u3e	M	156	2.92	0.216
1u3u	A	374	1.6	0.169
1u4g	A	298	1.4	0.18
1u5k	A	231	2	0.198
1u5m	A	73	N/A	
1u5p	A	200	2	0.187
1u6g	C	776	3.1	0.243
1u6k	A	266	1.55	0.178
1u7g	A	372	1.4	0.133
1u7h	A	333	1.8	0.158
1u7i	A	130	1.4	0.147
1u7k	A	129	1.85	0.21
1u7l	A	360	1.75	0.21
1u7p	A	163	1.9	0.19
1u84	A	81	1.6	0.205
1u8c	B	346	3.1	0.293
1u8v	A	490	1.6	0.163
1u98	A	269	2	0.2
1u9j	A	299	2.4	0.205
1u9l	B	68	1.9	0.216
1uaa	A	477	3	0.228
1uai	A	223	1.2	0.17
1uam	A	250	2.2	0.22
1uan	A	212	2	0.201
1uar	A	273	1.7	0.206
1ub1	A	125	N/A	
1ub4	C	62	1.7	0.21
1ubr	S	267	1.34	0.134
1ubw	A	320	2.5	0.192
1uc2	A	478	2.15	0.168
1uc6	A	60	N/A	
1ucp	A	91	N/A	
1ucr	B	75	1.2	0.138

1ucu	A	494	4	
1ud9	A	236	1.68	0.202
1udh	A	228	1.75	0.191
1udk	A	51	N/A	
1udl	A	98	N/A	
1udx	A	381	2.07	0.226
1ue0	A	176	2	0.203
1uea	D	158	2.8	0.197
1ueb	A	179	1.65	0.213
1uec	A	176	1.82	0.213
1uel	B	48	N/A	
1ufg	A	151	N/A	
1ufi	A	48	1.65	0.232
1ufk	A	247	1.9	0.232
1ufy	A	118	0.96	0.11
1ug0	A	88	N/A	
1ug1	A	92	N/A	
1ug4	A	56	1.6	0.239
1ugl	A	50	N/A	
1ugp	A	201	1.63	0.182
1ugq	B	228	2	0.184
1uh4	A	637	1.8	0.165
1uha	A	82	1.5	0.176
1uhc	A	79	N/A	
1uhr	A	93	N/A	
1uhu	A	105	N/A	
1uhw	A	109	N/A	
1ui0	A	192	1.5	0.183
1uix	A	68	1.8	0.193
1uiz	A	115	2.5	0.241
1uj2	A	213	1.8	0.19
1uj8	A	73	1.75	0.194
1ujl	A	42	N/A	
1ujs	A	88	N/A	
1ukf	A	188	1.35	0.204
1ukk	A	136	1.6	0.178
1ukx	A	137	N/A	
1uky	A	196	2.13	0.178
1ul5	A	86	N/A	
1ulb	A	289	2.75	0.204
1ulr	A	87	1.3	0.187
1uly	A	185	2.5	0.204
1um0	A	322	1.95	0.214
1um7	A	113	N/A	
1umg	A	359	1.8	0.183
1un1	A	239	2.1	0.206
1un2	A	171	2.4	0.206
1und	A	36	N/A	
1unl	D	150	2.2	0.216
1unn	D	112	1.9	0.179
1uon	A	1238	7.6	N/A
1uoy	A	64	1.5	0.164

1uoz	A	296	1.1	0.112
1upg	B	97	1.8	0.186
1upt	B	47	1.7	0.221
1uqr	K	151	1.7	0.178
1urj	B	968	3	0.235
1urk	A	130	N/A	
1urr	A	97	1.5	0.16
1us4	A	293	1.75	0.178
1us7	B	165	2.3	0.185
1usc	A	163	1.24	0.203
1uso	B	79	1.3	0.216
1usv	F	36	2.7	0.229
1ut1	A	140	1.7	0.173
1ut3	A	38	N/A	
1ut4	B	150	2.5	0.225
1ut8	B	271	2.75	0.235
1ute	A	302	1.55	0.213
1utx	A	66	1.9	0.153
1uuc	A	55	N/A	
1uuj	D	76	1.75	0.19
1uuq	A	402	1.5	0.121
1uv7	A	74	1.7	0.196
1uvy	A	104	2.4	0.211
1uw0	A	117	N/A	
1uw1	A	66	1.94	0.2
1uw2	A	61	N/A	
1uw4	C/D	91/245	1.95	0.204
1uw6	A	208	2.2	0.222
1uw7	A	104	2.8	0.228
1uwc	A	261	1.08	0.115
1uwf	A	158	1.69	0.179
1uwk	A	554	1.19	0.14
1uwv	A	407	1.95	0.186
1uww	A	181	1.4	0.189
1ux5	A	250	2.5	0.207
1uxa	A	185	1.5	0.18
1uxo	A	184	1.8	0.125
1uxy	A	340	1.8	0.202
1uz5	A	344	2.05	0.218
1uzb	A	516	1.4	0.177
1uzj	A	149	2.25	0.247
1uzv	A	106	1	0.115
1uzx	A	132	1.85	0.241
1v0d	A	131	2.6	0.218
1v0f	A	666	2.55	0.18
1v0j	A	386	2.25	0.225
1v18	B	26	2.1	0.207
1v1h	A	75	1.9	0.182
1v1m	B	333	2	0.129
1v2b	B	151	1.6	0.186
1v2x	A	183	1.5	0.225
1v30	A	118	1.4	0.217

1v31	A	93	N/A	
1v32	A	101	N/A	
1v33	A	346	1.8	0.219
1v3w	A	172	1.5	0.185
1v4a	A	406	2	0.228
1v4v	A	362	1.8	0.209
1v55	W	54	1.9	0.203
1v56	A	34	N/A	
1v5j	A	108	N/A	
1v5l	A	103	N/A	
1v5r	A	97	N/A	
1v5t	A	90	N/A	
1v64	A	108	N/A	
1v65	A	64	N/A	
1v6c	B	436	1.8	0.196
1v6g	A	81	N/A	
1v6s	A	390	1.5	0.201
1v6z	A	226	2	0.197
1v70	A	104	1.3	0.203
1v7b	A	175	1.9	0.208
1v7l	B	154	1.98	0.206
1v7o	A	128	2.62	0.235
1v7r	A	186	1.4	0.202
1v8c	A	165	1.6	0.221
1v8d	A	187	2.16	0.189
1v8h	A	106	1.2	0.211
1v8p	F	132	2.52	0.25
1v96	A	144	1.75	0.209
1v9l	A	358	2.8	0.2
1v9p	A	501	2.9	0.232
1v9x	A	114	N/A	
1vae	A	111	N/A	
1vaj	A	201	1.82	0.202
1vas	A	137	2.75	0.152
1vb5	B	275	2.2	0.209
1vc2	A	170	2.6	0.225
1vcc	A	77	1.6	0.218
1vdd	A	188	2.5	0.233
1vea	B	45	2.8	0.23
1vek	A	84	N/A	
1vf5	Q	34	3	0.258
1vf6	B	57	2.1	0.242
1vf7	E	145	2.4	0.258
1vfg	B	181	2.8	0.23
1vfr	A	208	1.8	0.187
1vfs	A	379	1.9	0.209
1vfy	A	67	1.15	0.174
1vg4	A	110	3.3	0.277
1vgg	A	159	1.75	0.18
1vgh	A	55	N/A	
1vgy	A	369	1.9	0.213
1vhe	A	354	1.9	0.176

1vhh	A	157	1.7	0.191
1vho	A	313	1.86	0.212
1vhv	B	246	1.75	0.211
1vhw	A	237	1.54	0.162
1vi0	A	184	1.65	0.231
1vi3	A	156	1.76	0.192
1vi4	A	156	1.87	0.229
1vi7	A	189	2.8	0.261
1vio	A	230	1.59	0.196
1vj2	A	114	1.65	0.208
1vj7	A	325	2.1	0.236
1vjn	A	188	2	0.178
1vjq	A	71	2.1	0.199
1vju	B	291	1.4	0.151
1vjv	A	359	1.74	0.179
1vk1	A	223	1.2	0.165
1vk5	A	121	1.6	0.16
1vk6	A	65	2.2	0.187
1vk8	C	91	1.8	0.142
1vkc	A	149	1.89	0.207
1vki	B	166	1.6	0.152
1vkm	E	282	1.9	0.154
1vkw	A	212	2	0.21
1vky	B	281	2	0.18
1vlo	A	348	1.7	0.159
1vlp	B	407	1.75	0.17
1vm0	B	99	1.8	0.174
1vma	B	294	1.6	0.207
1vmf	A	136	1.46	0.148
1vmg	A	80	1.46	0.142
1vp3	A	291	1.9	0.215
1vp6	A	133	1.7	0.19
1vp8	A	183	1.3	0.154
1vpb	A	415	1.75	0.178
1vpd	A	279	1.65	0.12
1vpm	A	149	1.66	0.17
1vpr	A	344	1.8	0.199
1vpu	A	45	N/A	
1vpz	A	54	2.05	0.218
1vqq	A	635	1.8	0.237
1vqr	A	272	2.25	0.193
1vqs	B	106	1.5	0.151
1vqu	A	314	1.85	0.179
1vqv	B	209	2.65	0.238
1vqz	A	316	1.99	0.159
1vr0	C	229	2.49	0.19
1vr3	A	161	2.06	0.162
1vr4	A	103	2.09	0.21
1vrn	L	272	2.2	0.191
1vrs	B	123	2.85	0.224
1vsg	A	362	2.9	0.22
1vtx	A	42	N/A	

1vve	A	118	N/A	
1vyi	A	110	1.5	0.173
1vyx	A	60	N/A	
1vz8	B	372	2.75	0.235
1vzm	A	39	1.4	0.192
1vzo	A	308	1.8	0.208
1vzs	A	76	N/A	
1vzy	A	277	1.97	0.197
1w0h	A	194	1.59	0.168
1w0j	G	52	2.2	0.184
1w0n	A	116	0.8	0.129
1w0u	A	55	1.8	0.22
1w1y	B	497	1.85	0.17
1w23	A	359	1.08	0.117
1w26	A	272	2.7	0.241
1w27	A	679	1.7	0.166
1w2b	O/R	138/72	3.5	0.192
1w2f	A	257	1.8	0.203
1w2w	A/N	207/190	1.75	0.174
1w2y	A	226	1.65	0.151
1w33	A	74	2.7	0.224
1w36	C	592	3.1	0.242
1w3d	A	46	N/A	
1w3f	A	276	2.58	0.231
1w4r	A	173	1.83	0.159
1w4x	A	533	1.7	0.212
1w53	A	83	1.6	0.198
1w5c	P/U/Y	179/100/359	3.2	N/A
1w5f	A	315	2	0.203
1w6s	C	595	1.2	0.158
1w70	A	56	1.46	0.181
1w7b	A	319	1.52	0.216
1w7j	A	747	2	0.194
1w7o	A	118	1.81	0.176
1w7p	B/D	148/152	3.6	0.292
1w8o	A	592	1.7	0.162
1w8x	N/P	114/80	4.2	N/A
1w94	A	148	2	0.198
1w9h	A	398	1.95	0.192
1wa9	B	63	3.15	0.228
1wap	A	68	1.8	0.178
1wb1	C	238	3	0.317
1wb4	A	281	1.4	0.111
1wb8	A	205	2.3	0.167
1wba	A	171	1.8	0.19
1wd5	A	205	2	0.195
1wd7	B	252	1.6	0.224
1wdd	W	120	1.35	0.16
1wdj	A	186	2	0.189
1we8	A	104	N/A	
1weh	A	169	1.8	0.185
1wem	A	76	N/A	

1weo	A	93	N/A	
1weq	A	85	N/A	
1wer	A	319	1.6	0.221
1weu	A	91	N/A	
1wew	A	78	N/A	
1wfk	A	88	N/A	
1wfm	A	138	N/A	
1wfp	A	74	N/A	
1wfr	A	143	N/A	
1wfw	A	74	N/A	
1wfx	A	175	2.8	0.22
1wfy	A	104	N/A	
1wfz	A	130	N/A	
1wg2	A	64	N/A	
1wg5	A	104	N/A	
1wgf	A	90	N/A	
1wgg	A	96	N/A	
1wgk	A	114	N/A	
1wgm	A	98	N/A	
1wgo	A	123	N/A	
1wgy	A	104	N/A	
1whb	A	157	N/A	
1whc	A	64	N/A	
1whf	A	73	N/A	
1whk	A	91	N/A	
1whm	A	92	N/A	
1whn	A	128	N/A	
1whq	A	99	N/A	
1whr	A	124	N/A	
1whs	B	144	2	0.175
1whv	A	100	N/A	
1wi0	A	113	N/A	
1wi8	A	104	N/A	
1wic	A	152	N/A	
1wif	A	126	N/A	
1wig	A	73	N/A	
1wij	A	127	N/A	
1wik	A	109	N/A	
1wim	A	94	N/A	
1win	A	143	N/A	
1wip	A	363	4	0.452
1wix	A	164	N/A	
1wiz	A	101	N/A	
1wj4	A	124	N/A	
1wj9	A	188	1.9	0.228
1wjj	A	145	N/A	
1wjp	A	107	N/A	
1wjr	A	127	N/A	
1wjw	A	112	N/A	
1wk1	A	150	N/A	
1wkc	A	166	1.7	0.218
1wkr	A	340	1.3	0.147

1wkt	A	88	N/A	
1wlh	A	260	2.8	0.257
1wlq	C	142	2.8	0.231
1wm2	A	78	1.6	0.169
1wmh	B	82	1.5	0.216
1wmt	A	41	N/A	
1wnh	A	210	1.83	0.211
1wo9	A	35	N/A	
1woh	A	303	1.75	0.219
1woq	A	253	1.8	0.193
1wot	A	98	N/A	
1wp1	A	33	2.56	0.252
1wpb	A	159	2	0.19
1wpd	A	34	N/A	
1wpg	A	612	2.3	0.223
1wpn	A	177	1.3	0.139
1wpx	B	185	2.7	0.183
1wq8	A	99	1.9	0.192
1wqj	B	80	1.6	0.184
1wri	A	93	1.2	0.109
1ws7	A	107	1.9	0.188
1wte	A	272	1.9	0.171
1wty	A	114	2.2	0.23
1wu3	I	153	2.15	0.192
1wu9	B	67	1.54	0.181
1wub	A	176	1.65	0.219
1wuf	A	340	2.9	0.236
1wvi	A	250	2.3	0.248
1www	W	103	2.2	0.189
1wxi	A	261	1.7	0.176
1wy2	A	351	1.7	0.187
1x6i	A	86	1.2	0.171
1x74	A	354	1.79	0.16
1x7f	A	330	2.3	0.171
1x7o	A	261	2.37	0.206
1x82	A	183	1.5	0.161
1x99	A	141	1.4	0.174
1x9a	A	87	N/A	
1x9n	A	624	3	0.235
1x9y	A	323	2.5	0.244
1xak	A	68	1.8	0.223
1xb2	B	234	2.2	0.218
1xbn	A	195	2.5	0.263
1xbr	A	176	2.5	0.215
1xc0	A	33	N/A	
1xcb	A	204	2.9	0.228
1xcg	E	358	2.5	0.224
1xd3	A	215	1.45	0.166
1xd4	A	72	3.64	0.307
1xdn	A	262	1.2	0.128
1xe0	A	108	1.7	0.208
1xex	B	118	2.5	0.207

1xfd	A	236	3	0.248
1xfi	A	343	1.7	0.169
1xfs	A	149	1.7	0.221
1xg0	B	67	0.97	0.107
1xg8	A	108	2.1	0.229
1xhj	A	88	N/A	
1xho	C	106	2.2	0.194
1xi7	A	47	N/A	
1xik	B	331	1.7	0.206
1xiw	B	64	1.9	0.204
1xiz	B	154	2	0.203
1xjh	A	62	N/A	
1xjt	A	170	1.75	0.208
1xjv	A	287	1.73	0.228
1xkb	A	90	2.4	0.206
1xki	A	122	1.8	0.189
1xkr	A	205	1.75	0.226
1xkt	A	166	2.6	0.258
1xl6	A	162	2.85	0.27
1xl8	B	568	2.2	0.199
1xly	B	156	1.95	0.191
1xmq	M	26	3	0.222
1xmw	A	178	N/A	
1xmx	A	380	2.1	0.168
1xod	B	116	1.15	0.154
1xp0	A	326	1.79	0.193
1xpa	A	113	N/A	
1xpv	A	78	N/A	
1xqb	A	161	2.85	0.279
1xqo	A	253	1.03	0.158
1xqs	D	53	2.9	0.237
1xrg	B	136	2.2	0.193
1xri	A	141	3.3	0.204
1xrs	B	195	2.8	0.199
1xs0	C	129	1.58	0.225
1xs5	A	237	1.85	0.183
1xs9	D	81	N/A	
1xsk	A	766	2.2	0.235
1xsl	A	308	2.3	0.207
1xso	A	150	1.49	0.104
1xsz	A	328	1.41	0.202
1xte	A	116	1.6	0.187
1xu4	A	296	2.4	0.196
1xuu	A	348	1.9	0.207
1xv3	A	47	N/A	
1xwe	A	151	N/A	
1xwh	A	66	N/A	
1xwv	A	127	1.83	0.221
1xxa	C	72	2.2	0.2
1xxe	A	268	N/A	
1xyz	A	320	1.4	0.183
1xzp	A	336	2.3	0.226

1xzz	A	213	1.8	0.205
1y02	A	91	1.8	0.206
1y0u	A	86	1.6	0.172
1y12	C	155	1.95	0.179
1y13	A	161	2.2	0.195
1y1v	A	914	3.8	0.282
1y1x	A	182	1.95	0.187
1y23	E	141	2.3	0.213
1y2i	A	106	2.3	0.192
1y2s	A	113	N/A	
1y4c	A	466	1.9	0.202
1y51	A	87	1.65	0.205
1y60	D	165	1.9	0.209
1y67	D	214	1.85	0.184
1y6d	A	114	N/A	
1y6x	A	86	1.25	0.183
1y6z	B	234	1.88	0.206
1y71	B	108	1.95	0.214
1y7j	A	40	N/A	
1y7p	B	216	1.9	0.211
1y7q	A	98	N/A	
1y7r	A	126	1.7	0.172
1y7y	A	69	1.69	0.169
1y88	A	184	1.85	0.202
1y8c	A	124	2.5	0.221
1y8r	E	356	2.75	0.22
1y8t	A	273	2	0.227
1y9i	A	158	1.8	0.211
1y9j	A	140	N/A	
1y9m	A	517	1.89	0.165
1y9q	A	173	1.9	0.199
1y9w	A	137	1.9	0.202
1yb2	A	213	2.01	0.199
1yb3	A	163	1.6	0.204
1yb5	A	324	1.85	0.17
1ybt	C	140	2.31	0.194
1ybx	B	88	1.8	0.225
1ybz	A	76	1.82	0.201
1yc9	A	408	1.8	0.189
1ycn	A	278	2.51	0.22
1yd2	A	90	1.6	0.175
1ydl	A	68	2.3	0.236
1yea	A	107	1.9	0.19
1yez	A	68	N/A	
1yf2	A	185	2.4	0.236
1yg2	A	137	2.2	0.233
1ygb	A	448	2.48	0.199
1yge	A	821	1.4	0.197
1ygp	A	858	2.8	0.179
1ygt	A	104	1.7	0.193
1ygy	A	520	2.3	0.205
1yhu	S	105	3.15	0.243

1yis	A	403	2.4	0.174
1yle	A	320	1.7	0.19
1ylm	A	139	1.83	0.2
1ylq	A	93	2.02	0.217
1yn3	A	98	1.35	0.218
1yn4	A	99	1.8	0.233
1yni	A	440	2.2	0.219
1yoy	A	142	2	0.184
1yoz	B	115	2	0.231
1ypy	A	182	1.51	0.239
1yua	A	122	N/A	
1yui	A	54	N/A	
1yv5	A	336	2	0.205
1yw4	A	315	2	0.221
1yy7	A	206	2.02	0.179
1z0r	A	53	N/A	
1zdb	A	38	N/A	
1zrr	A	179	N/A	
2abk	A	211	1.85	0.185
2abl	A	163	2.5	0.183
2ach	B	35	2.7	0.181
2adr	A	60	N/A	
2alc	A	65	N/A	
2ame	A	66	2	0.185
2atc	B	152	3	0.27
2b82	A	211	1.25	0.165
2bb8	A	71	N/A	
2bbi	A	71	N/A	
2bbk	L	124	1.75	0.167
2bbv	C	321	2.8	0.221
2ben	A	170	1.8	0.217
2beq	D	46	1.6	0.199
2bez	F	31	1.6	0.206
2bf9	A	35	0.99	0.137
2bg5	A	307	1.82	0.208
2bg9	C/E	369/370	4	N/A
2bgz	A	293	0.12	N/A
2bh8	B	87	1.9	0.192
2bhg	A	184	1.9	0.218
2bhn	A	14	3.2	0.216
2bid	A	197	N/A	
2bk6	C	154	2.19	0.211
2bn2	A	85	2.8	0.208
2bpa	3-Jan	426/36	3	0.209
2bs2	C	206	1.78	0.229
2btv	B/T	844/256	3.5	0.266
2c8s	A	138	1.6	0.21
2cb5	A	452	1.85	0.182
2cbh	A	36	N/A	
2ccy	A	125	1.67	N/A
2ci2	I	65	2	0.198
2cjn	A	97	N/A	

2cpo	A	298	2.1	0.186
2dpm	A	244	1.8	0.238
2dvh	A	23	N/A	
2erl	A	40	1	0.129
2evn	A	103	N/A	
2fcb	A	170	1.74	0.194
2fha	A	172	1.9	0.186
2g3p	A	196	1.9	0.26
2gep	A	456	1.9	0.175
2gli	A	155	2.6	0.228
2gmf	A	107	2.4	0.235
2gpr	A	152	2.5	0.186
2gva	A	87	N/A	
2hdh	A	280	2.2	0.198
2hmx	A	133	N/A	
2hqi	A	72	N/A	
2ifo	A	46	N/A	N/A
2ilk	A	153	1.6	0.163
2isb	A	175	1.66	0.174
2isd	B	493	2.5	0.227
2jhb	A	143	N/A	
2jqa	A	113	N/A	
2kfn	A	511	2.03	0.214
2lal	B	47	1.8	0.189
2lis	A	131	1.35	0.136
2mas	A	300	2.3	0.205
2mcm	A	112	1.5	0.153
2mcp	H	222	3.1	0.185
2mob	A	94	N/A	
2mrb	A	31	N/A	
2nbt	A	66	N/A	
2nef	A	136	N/A	
2nlr	A	206	1.2	0.111
2occ	D/I/U/X	134/68/60/32	2.3	0.209
2opo	C	77	1.75	0.198
2orc	A	71	N/A	
2paq	A	171	2.1	0.187
2pda	A	1231	3	0.234
2pk8	A	94	1.85	0.207
2plc	A	256	2	0.198
2prg	C	17	2.3	0.207
2pth	A	193	1.2	0.196
2pvb	A	107	0.91	0.11
2rel	A	57	N/A	
2rmc	A	181	1.64	0.197
2tgf	A	50	N/A	
2tnf	A	142	1.4	0.201
2utg	A	70	1.64	0.19
2vsg	A	342	2.7	0.203
2wbc	A	183	2.3	0.187
3c2c	A	112	1.68	0.175
3eip	A	84	1.8	0.218

3eng	A	213	1.9	0.145
3kin	B	117	3.1	0.289
3lri	A	83	N/A	
3nul	A	125	1.6	0.181
3pcf	R	233	2.15	0.179
3por	A	288	2.5	0.188
3rhn	A	114	2.1	0.194
3sli	A	678	1.8	0.187
3ssi	A	107	2.3	0.185
487d	K	148	7.5	N/A
4bcl	A	349	1.9	0.178
4dpv	Z	532	2.9	0.283
4hb1	A	44	2.9	0.234
4mt2	A	61	2	0.197
4pro	D	143	2.4	0.191
4sgb	I	51	2.1	0.142
4tgl	A	265	2.6	0.129
4ubp	A	100	1.55	0.151
5acn	A	753	2.1	0.215
5gat	A	66	N/A	
5hpg	A	84	1.66	0.166
5r1r	A	637	3.1	0.196
7msf	A	123	2.8	0.2
8fab	A/B	204/204	1.8	0.173
8prn	A	287	2.3	0.177

* Indicates number of residues in PDB for which torsion angles were calculated with the SECSTR program.
N/A indicates that the value was not available.
NMR derived structures have N/A in Resolution column and no entry in the R-factor column.
Only protein fragments with all main-chain atoms having B-factor ≤ 60 ($Å^2$) were selected for clustering.
