# Supplementary material for: Functionally important segments in proteins dissected using Gene Ontology and geometric clustering of peptide fragments
Source: Genome Biol. 2008 Mar 10;9(3):R52. doi: 10.1186/gb-2008-9-3-r52 (PMC2397504; doi:10.1186/gb-2008-9-3-r52)
Supplement: Additional data file 2 — Distribution of compactness values for FL8 and FL16 clusters. [file gb-2008-9-3-r52-S2.pdf]

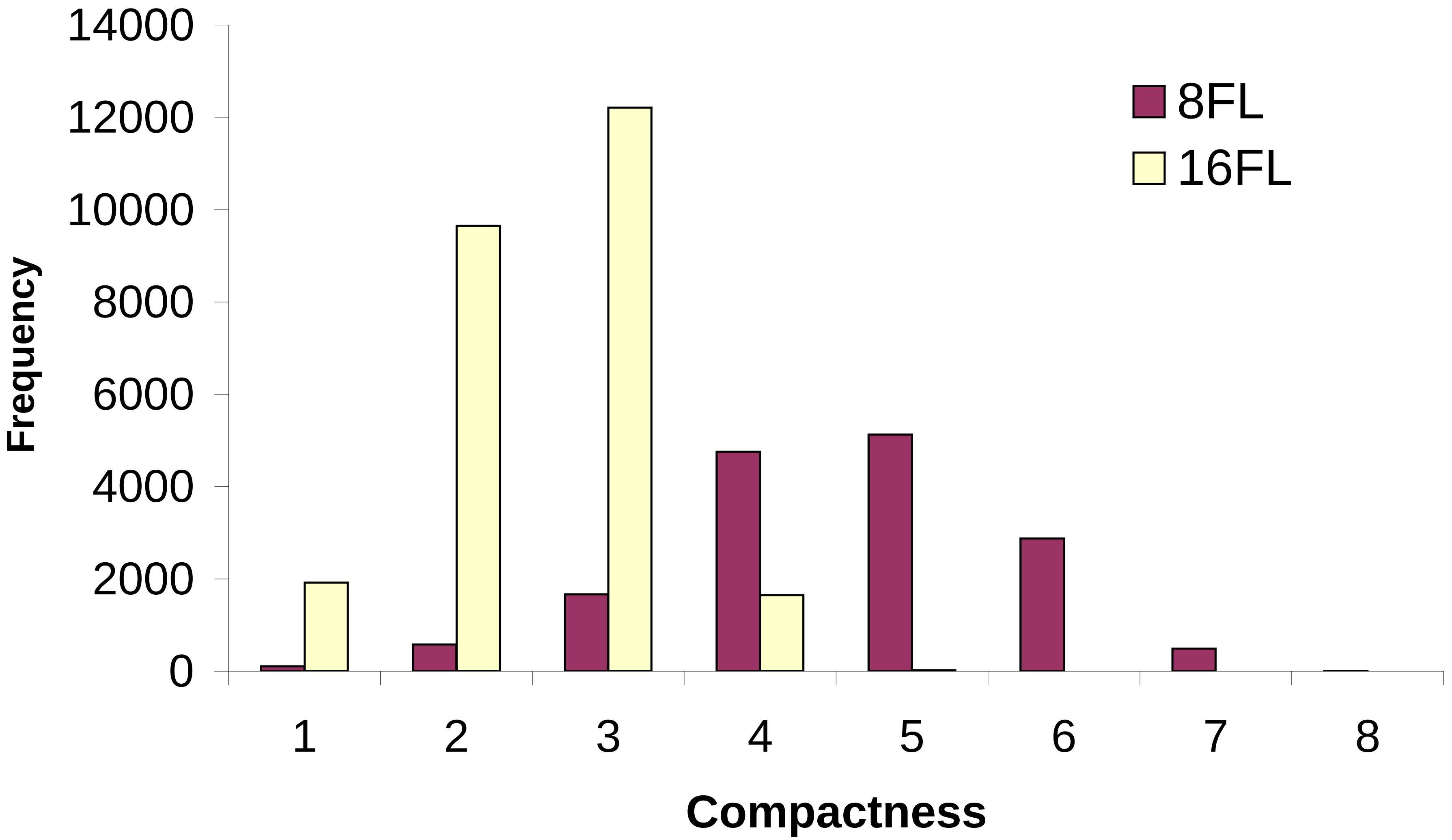

Supp. Fig. 1. Histogram showing the distribution of compactness values for 8 and 16 fragment length clusters. Bin 1 in X axis refers to values between 0 to 1 and so forth.
